# Effects of agrochemical pollution on schistosomiasis transmission: a systematic review and modelling analysis

**DOI:** 10.1016/S2542-5196(20)30105-4

**Published:** 2020-07

**Authors:** Christopher M Hoover, Samantha L Rumschlag, Luke Strgar, Arathi Arakala, Manoj Gambhir, Giulio A de Leo, Susanne H Sokolow, Jason R Rohr, Justin V Remais

**Affiliations:** Division of Environmental Health Sciences, Berkeley School of Public Health, University of California, Berkeley, Berkeley, CA, USA; Department of Biological Sciences, Eck Institute of Global Health, University of Notre Dame, Notre Dame, IN, USA; Division of Environmental Health Sciences, Berkeley School of Public Health, University of California, Berkeley, Berkeley, CA, USA; Discipline of Mathematics, School of Sciences, Royal Melbourne Institute of Technology University, Melbourne, VIC, Australia; Port Melbourne, VIC, Australia; Department of Biology, Hopkins Marine Station, Stanford University, Pacific Grove, CA, USA; Woods Institute for the Environment and Center for Innovation in Global Health, Stanford University, Stanford, CA, USA; Department of Biology, Hopkins Marine Station, Stanford University, Pacific Grove, CA, USA; Woods Institute for the Environment and Center for Innovation in Global Health, Stanford University, Stanford, CA, USA; Department of Biological Sciences, Eck Institute of Global Health, University of Notre Dame, Notre Dame, IN, USA; Division of Environmental Health Sciences, Berkeley School of Public Health, University of California, Berkeley, Berkeley, CA, USA

## Abstract

**Background:**

Agrochemical pollution of surface waters is a growing global environmental challenge, especially in areas where agriculture is rapidly expanding and intensifying. Agrochemicals might affect schistosomiasis transmission through direct and indirect effects on *Schistosoma* parasites, their intermediate snail hosts, snail predators, and snail algal resources. We aimed to review and summarise the effects of these agrochemicals on schistosomiasis transmission dynamics.

**Methods:**

We did a systematic review of agrochemical effects on the lifecycle of *Schistosoma* spp and fitted dose-response models to data regarding the association between components of the lifecycle and agrochemical concentrations. We incorporated these dose-response functions and environmentally relevant concentrations of agrochemicals into a mathematical model to estimate agrochemical effects on schistosomiasis transmission. Dose-response functions were used to estimate individual agrochemical effects on estimates of the agrochemically influenced basic reproduction number, *R*_0_, for *Schistosoma haematobium*. We incorporated time series of environmentally relevant agrochemical concentrations into the model and simulated mass drug administration control efforts in the presence of agrochemicals.

**Findings:**

We derived 120 dose-response functions describing the effects of agrochemicals on schistosome lifecycle components. The median estimate of the basic reproduction number under agrochemical-free conditions, was 1·65 (IQR 1·47–1·79). Agrochemical effects on estimates of *R*_0_ for *S haematobium* ranged from a median three-times increase (*R*_0_ 5·05, IQR 4·06–5·97) to transmission elimination (*R*_0_ 0). Simulations of transmission dynamics subject to interacting annual mass drug administration and agrochemical pollution yielded a median estimate of 64·82 disability-adjusted life-years (DALYs) lost per 100,000 people per year (IQR 62·52–67·68) attributable to atrazine use. In areas where aquatic arthropod predators of intermediate host snails suppress transmission, the insecticides chlorpyrifos (6·82 DALYs lost per 100,000 people per year, IQR 4·13–8·69) and profenofos (103·06 DALYs lost per 100,000 people per year, IQR 89·63–104·90) might also increase the disability burden through their toxic effects on arthropods.

**Interpretation:**

Expected environmental concentrations of agrochemicals alter schistosomiasis transmission through direct and indirect effects on intermediate host and parasite densities. As industrial agricultural practices expand in areas where schistosomiasis is endemic, strategies to prevent increases in transmission due to agrochemical pollution should be developed and pursued.

## Introduction

More than 200 million people globally are affected by schistosomiasis, which is caused by parasitic trematodes of the genus *Schistosoma*. Schistosome parasites have a complex lifecycle in freshwater, rendering them sensitive to the physical, chemical, and biological conditions of the aquatic environment. Schistosomiasis transmission is linked to agricultural expansion, particularly water resource development, such as dam construction and irrigation projects, which can expand the suitable habitat for intermediate host snails and can affect the distribution of predators capable of suppressing snail populations.^1–3^ In these same environments, agrochemical pollution might cause similar ecological disruptions that increase snail resources, kill snail predators, or affect schistosomes directly, but the effects of agrochemicals on schistosomiasis transmission have not been systematically investigated.^4^

Schistosomiasis-endemic regions of sub-Saharan Africa, where more than 90% of schistosomiasis cases occur, have historically had low agrochemical use, owing to the predominance of small-scale farming.^5^ However, global agrochemical use is increasing quickly as agrochemical inputs become more readily available and as developing economies rely on less labour-intensive methods of agricultural production. In schistosomiasis endemic areas of sub-Saharan Africa, the scarcity of local production of agrochemicals has suppressed their widespread application.^6^ However, the completion of the Indorama fertiliser plant in Nigeria and the planning of additional plants in Ethiopia and Rwanda are expected to increase agrochemical access and use.^5–9^

**Figure 1:**
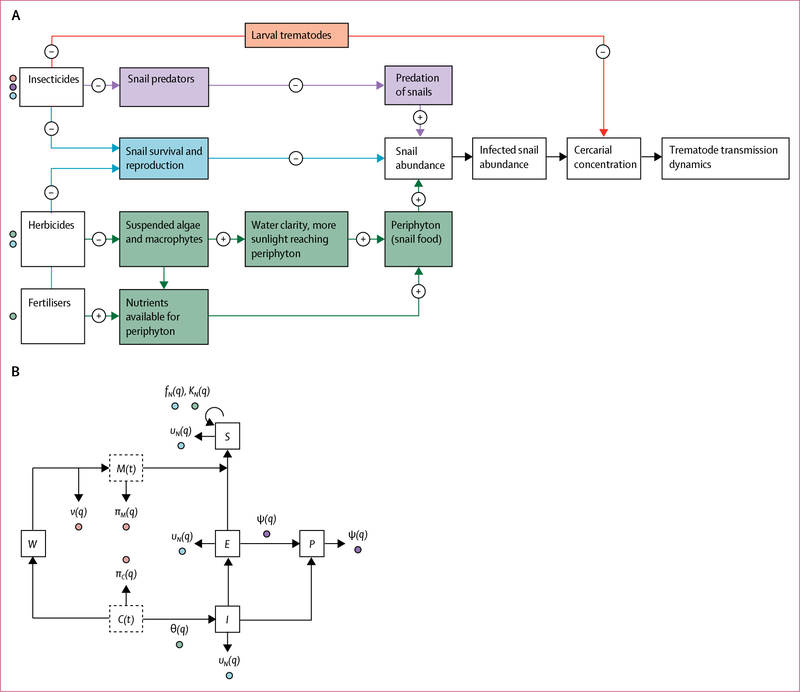
Summary of agrochemical effects on the schistosome lifecycle and translation into a dynamic model of schistosomiasis transmission Shown are the pathways through which different classes of agrochemicals may affect schistosomiasis transmission dynamics (A). Colours indicate the type of effect: green represents bottom-up stimulation of algal resources that benefit the intermediate host snail population, blue represents direct toxicity to snails, purple represents top-down effects on the intermediate host snail population through toxicity to snail predators, and red represents direct toxicity to trematode larvae and eggs. These effects are incorporated into the model (B) through model parameters that are a function of agrochemical concentration, *q*. Model parameters affected by agrochemicals include schistosome egg viability (model parameter, *v*), miracidial survival (π*_M_*), cercarial survival (π*_C_*), snail cercarial shedding (θ), snail fecundity (*f_N_*), snail mortality (μ*_N_*), snail environmental carrying capacity (*K_N_*), predator mortality (μ*_p_*), and predator consumption rate of snails (ψ).

A large body of published literature has shown that agrochemicals affect the transmission of non-human trematodes through direct effects on parasites and intermediate snail hosts and through indirect trophic cascades.^10–15^ Fertilisers and herbicides trigger bottom-up trophic cascades by altering algal dynamics to benefit periphytic algae, a key food resource for snail populations.^11,12,16^ Insecticides cause top-down trophic cascades, whereby snails are released from predation due to the high toxicity of insecticides to aquatic arthropods that prey on snails.^2,10,17^ Additionally, all three types of agrochemical directly affect snail survival and reproduction, schistosome egg viability, cercarial survival, and miracidial survival (figure 1).^10,15,18^ Evidence suggests that certain agrochemicals can increase the risk of human schistosomiasis,^10^ but the array of agrochemical effects on human schistosomiasis transmission has not been systematically investigated. We therefore aimed to review and summarise the effects of these agrochemicals on schistosomiasis transmission dynamics.

## Methods

### Search strategy and selection criteria

We reviewed published literature examining agrochemical effects on parameters that govern transmission of schistosomes (appendix p 2) and other trematodes in Web of Science and SCOPUS. For parameters representing cercarial survival, miracidial survival, schistosome egg viability, snail reproduction, snail survival, cercarial shedding rate, and snail resource availability, we used the schistosome-related and snail-related search terms “schistosom*”, “cercariae”, “miracidia”, “biomphalaria”, “bulinus”, and “oncomelania” (intermediate host snail genera for the three main *Schistosoma* species) and the agrochemical-related search terms “fertiliser”, “pesticide”, “herbicide”, “fungicide”, and “insecticide”. For parameters representing snail predator survival and predation rates, these same agrochemical search terms were used in conjunction with the search terms “procambarus” (crawfish genus), “macrobrachium” (prawn genus), and “belostoma” (waterbug genus). Manuscripts from these searches that included quantitative data relating agrochemical concentration to rates (eg, mortality) or other aspects (eg, egg viability and carrying capacity) in the system that could inform model parameters were included. Categorised studies were organised in a reference library. Data were extracted directly from published tables or from plots using Plot Digitizer, and dose-response functions were fitted.

### Mathematical model

We extended previously published dynamic transmission models^10,19–22^ for helminth infections to incorporate parameters sensitive to agrochemical concentrations (figure 1B). Infection dynamics of the intermediate host snail population were simulated in a susceptible-exposed-infected framework. Human infection was modelled as the negative binomially distributed population mean parasite burden. A dynamic predator population that feeds on intermediate host snails at a density-dependent rate estimated by Holling’s disc equation was also included.^2,17,21,23^ The model was fitted to *Schistosoma haematobium* infection data collected from an ongoing study.^10^ Additional details of the model and epidemiological data including parameterisation, fitting procedure, and model equations are provided in the appendix (pp 5–10).

### Dose-response functions

Agrochemical effects on model parameters were expressed as dose-response functions as follows: for each parameter, *p*, found to respond to concentration, *q_c_*, of agrochemical, *c,* a function relating the parameter value is fitted as *p_jc_*(*q_c_*)=*Pf_jc_*(*q_c_*), where *P* is the parameter’s agrochemical-free value (appendix p 7), and *f_jc_*(*q_c_*) is a function derived from data reported in study, *j*, that quantifies relative changes in the parameter to agrochemical concentration, *q_c_*. Studies that directly reported parameters of a fitted dose-response function (eg, an LC_50_ and slope parameter from the Litchfield and Wilcoxon method^24^) were included following derivation of *f_jc_*(*q_c_*) from reported results (appendix p 4).

The set of model parameters comprising *p* and exhibiting sensitivity to agrochemical concentrations include schistosome egg viability (model parameter, *v*), miracidial survival (π*_M_*), cercarial survival (*π_C_*), snail cercarial shedding (θ), snail fecundity (*f_N_*), snail mortality (μ*_N_*), snail environmental carrying capacity (*K_N_*), predator mortality (μ*_p_*), and predator consumption rate of snails (ψ).

### Environmentally relevant concentrations

Agrochemical concentrations in surface waters are affected by application amount and frequency, chemical properties that affect mobility and persistence, and environmental conditions that determine transport. To address the limited monitoring of agrochemicals in sub-Saharan Africa^5^ we drew on both modelled and observed sources to determine environmentally relevant surface water concentrations.^25^ We used the Pesticide in Water Calculator, software used by the US Environmental Protection Agency and Health Canada to model the fate and transport of agrochemicals, to generate peak expected environmental concentrations (EEC*_c_*) based on pesticide traits, applications, and soil and climatic characteristics (appendix p 4).^26^ Additionally, data from three of the most comprehensive pesticide monitoring databases in the USA were used to determine peak observed concentrations (POC*_c_*; appendix p 4).

### Modelled effects on schistosomiasis transmission

An analytic expression of the basic reproduction number as a function of agrochemical concentration, denoted *R*_0_(*q_c_*), was derived from the model using the next generation matrix method (appendix pp 8–10). We used *R*_0_(*q_c_*) as a steady-state summary of transmission intensity within a fully susceptible human population to compare effects across studies, agrochemicals, and parameters. Component effects capturing the influence of single dose-response functions, *R*_0_*_jc_*(*q_c_*), and net effects representing the influence of multiple dose-response functions driven by the same agrochemical, *R*_0_*_c_*(*q_c_*), were estimated.

The model was simulated through time under different agrochemical pollution and intervention scenarios to estimate disability-adjusted life-years (DALYs) lost per 100,000 people per year due to agrochemically altered *S haematobium* infection. Previously published estimates of disability weights associated with heavy (≥50 eggs per mL urine) or light (>0–<50 eggs per mL urine) infection with *S haematobium* were used with modelled egg burden distributions to estimate disability as described previously.^21,27^

To estimate component agrochemical effects on transmission at environmentally relevant concentrations, each dose-response function, *p_jc_*(*q_c_*), is incorporated into the *R*_0_(*q_c_*) expression while holding all other parameters at their agrochemical-free values, *P*. Peak EEC*_c_* values from Pesticide in Water Calculator simulations were used to estimate *R*_0_*_jc_*(EEC_c_)—ie, an estimate of the basic reproductive rate influenced only by the agrochemical effect of *p_jc_*(EEC_c_). Uncertainty associated with model fitting to epidemiological data and fitting dose-response functions to agrochemical data is incorporated into estimates of *R*_0_*_jc_*(EEC_c_) with Monte Carlo simulation with 1000 random draws of a weighted sample of the best fit transmission parameters and of *p_jc_*(*q_c_*) to generate a distribution of *R*_0_*_jc_*(EEC_c_) estimates.

To determine emergent properties of multiple response functions acting simultaneously, we estimated the net effect of agrochemicals acting on multiple parameters by estimation of *R*_0_*_c_*(*q_c_*). For chemicals with dose-response functions identified for all hypothesised effects, *R*_0_*_c_*(*q_c_*) was estimated across a range of concentrations from 0–2 × EEC_c_. These estimates incorporate all dose-response functions identified in the review for chemical, *c*, into the *R*_0_*_c_*(*q_c_*) expression. Uncertainty associated with model and dose-response function fit was propagated to *R*_0_*_c_*(*q_c_*) with the aforementioned Monte Carlo simulation.

To explore the temporal dynamics of schistosomiasis transmission in the presence of agrochemical pollution, we incorporated time series of agrochemical concentrations generated from Pesticide in Water Calculator into model simulations of an annual mass drug administration (MDA) campaign with praziquantel administered at 80% coverage and 93% efficacy, based on previous work.^28^ Because predator populations are not commonly considered in models of schistosomiasis control and have probably been extirpated in many areas with high schistosomiasis transmission,^29^ we simulated MDA in scenarios both with and without predators. Simulations with and without agrochemical influence were run to estimate DALYs attributable to agrochemical pollution (appendix p 8). Simulations were run 1000 times using the R package deSolve (version 1.27.1)^30^ to incorporate uncertainty in model and dose-response function fit.

### Role of the funding source

The funders of the study had no role in study design, data collection, data analysis, data interpretation, or writing of the report. The authors had full access to all the data in the study and had final responsibility for the decision to submit for publication.

## Results

From 852 studies identified in the review, a total of 144 quantitative associations between agrochemicals and parameters of the schistosomiasis transmission model were identified from 47 different studies (table 1; appendix p 3). From these, 120 dose-response functions were fit (appendix pp 1–2), whereas 24 experiments had insufficient data to fit a dose-response curve, because they compared a control group with a single agrochemical treatment group. Such experiments were included in the estimation of component effects by estimating *R*_0_*_jc_*(*q_c_*) at the concentration of the single treatment group in study *j*, but were not included in simulations requiring full response functions.

**Figure 2:**
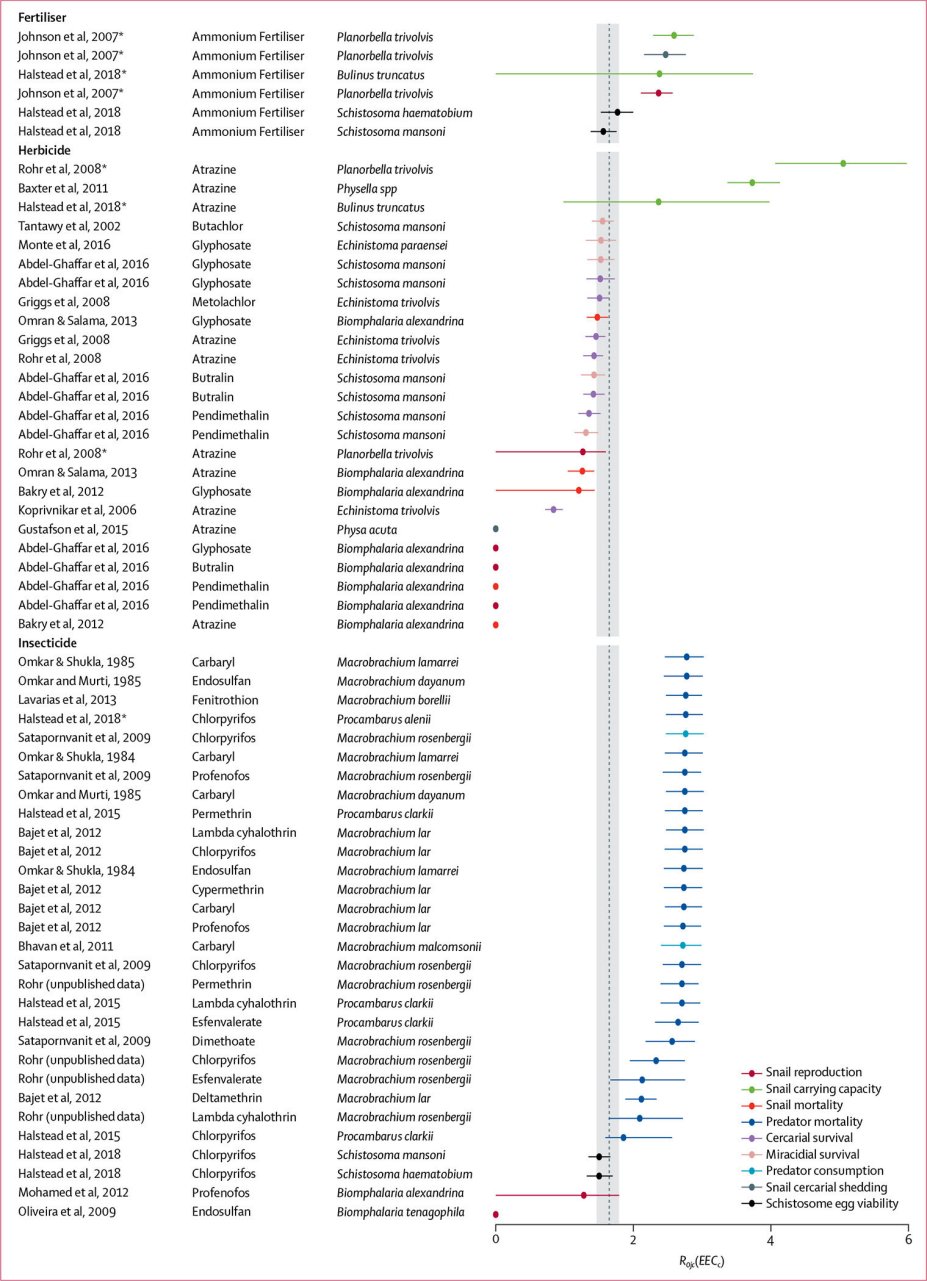
Forest plot displaying component agrochemical effects on *R*_0_*_jc_*(EEC_c_) Studies are listed by reference, agrochemical, and species corresponding to an experiment from which a dose-response function was estimated. The forest plot shows the median and IQR of the distribution of the agrochemical’s effect on the basic reproductive number, *R*_0_*_jc_*(EEC_c_), produced in 1000 Monte Carlo simulations drawn from the best fit transmission parameters of the epidemiological model and from each agrochemical dose-response function at *p_jc_*(EEC_c_). Results are divided by agrochemical type (fertilisers, herbicides, and insecticides), and the vertical solid line and shaded region indicates the median and IQR of agrochemical-free estimates of *R*_0_(*q_c_*=0) 1·65 (IQR 1·47–1·79). Colours indicate the parameter affected, as indicated in the legend. EEE_c_=expected environmental concentration of agrochemical c. *p_jc_*(EEC_c_)*=*the parameter value estimated from a dose-response function derived from study j for chemical c at its EEC. *R*_0_*_jc_*(EEC_c_)*=*the basic reproduction number estimated when incorporating a dose-response function from study j at chemical c’s EEC. *R*_0_*_jc_*(*q_c_*)*=*the basic reproduction number estimated when incorporating agrochemical effects on a parameter at the concentration tested in the experiment, q*_c_*, for studies that only compared an agrochemical treatment group to a control group. *Studies reported as *R*_0_*_jc_*(*q_c_*) for the concentration tested in the experiment, rather than EEC_c_, as the experiment compared a control group to an agrochemical group at a single concentration, and thus there was insufficient data to fit a full dose-response function. Only dose-response functions that produce *R*_0_*_jc_*(EEE_c_) plus or minus 5% from the median baseline, *R*_0_(*q_c_*=0), are shown for figure clarity.

The median estimate of the basic reproduction number under agrochemical-free conditions, *R*_0_(*q_c_*=0), was 1·65 (IQR 1·47–1·79). Experiments investigating the effects of fertiliser suggest increases in transmission due to bottom-up effects that increase snail carrying capacity, snail reproductive rates, and cercarial shedding rates (figure 2). Herbicides, particularly atrazine and glyphosate, also increase transmission at their EEC_c_ through bottom-up effects that increase snail carrying capacity, but decrease transmission due to direct effects that decrease cercariae and miracidia survival, snail reproduction, and snail survival (figure 2). A variety of insecticides, including chlorpyrifos, profenofos, cypermethrin, permethrin, deltamethrin, λ-cyhalothrin, esfenvalerate, carbaryl, and dimethoate, increased mortality of snail predators to increase transmission through top-down effects at their EEC_c_ (figure 2). Many of these effects are sufficient to extirpate the predator population, leading to *R*_0_(EEC_c_) estimates equivalent to those estimated in a predator-free model (*R*_0_*_,Pred-Free_* 2·72, IQR 2·45–2·99). There is also evidence that insecticides decrease transmission through direct effects on cercariae and miracidia survival, snail reproduction, and snail survival, although only the effects of profenofos and endosulfan on snail reproduction produced estimates of *R*_0_*_jc_*(EEC_c_) that differed from baseline estimates (figure 2).

**Figure 3:**
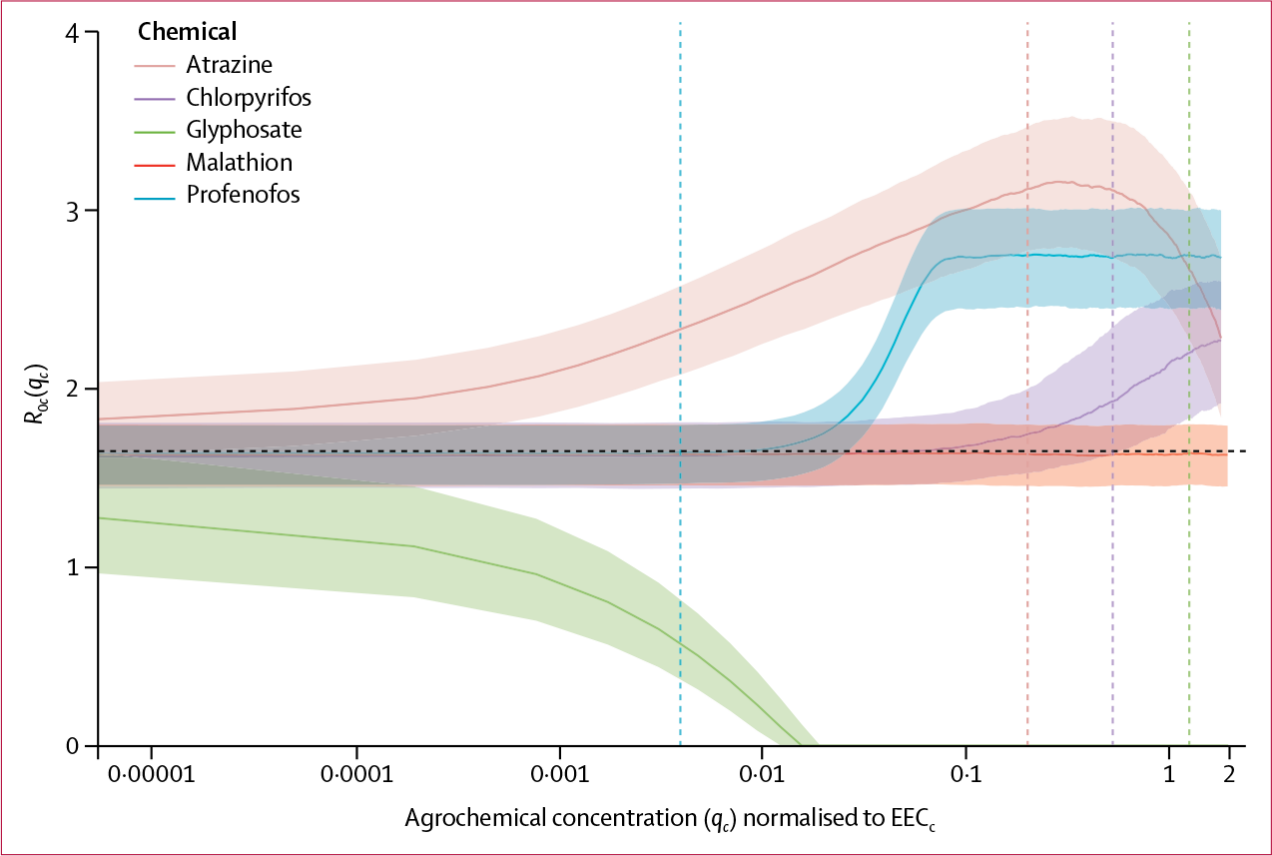
Net responses of *R*_0_*_c_(q_c_)* to herbicide and insecticide concentrations normalised to peak EEC_c_ The solid lines represent median basic reproduction number *R*_0_*_c_*(*q_c_*) estimates and shaded regions indicate the IQR of 1000 Monte Carlo simulations. The horizontal dashed line represents the baseline, agrochemical-free estimate, of the basic reproduction number *R*_0_(*q_c_*=0)=1·65. Vertical dashed lines indicate each chemical’s peak observed concentration (POC_c_) across the monitoring databases, and agrochemical concentration on the x-axis is normalised to each chemical’s peak expected environmental concentration (EEC_c_).

Dose-response functions were estimable across all parameters affected by the insecticides malathion, chlorpyrifos, and profenofos and the herbicides atrazine and glyphosate, and thus the net effects of these five agrochemicals on transmission were estimated by incorporating multiple dose-response functions acting 0 simultaneously into *R*_0_*_c_*(*q_c_*). Amongthe threeinsecticides investigated across multiple parameters, malathion did not have a significant net effect on *R*_0_*_c_*(*q_c_*) at environmentally relevant concentrations (*R*_0_*_,malathion_*[EEC_malathion_] 1·63, IQR 1·45–1·81; figure 3). However, both chlorpyrifos (*R*_0_*_,chlorpyrifos_*[EEC_chlorpyrifos_] 2·12, IQR 1·73–2·52) and profenofos (*R*_0_*_,profenofos_*[EEC_profenofos_] 2·75, IQR 2·44–3·01) were found to substantially amplify transmission due to their toxicity to snail predators, an indirect top-down effect (figure 3). Whereas the high toxicity of profenofos to snail predators^31^ yielded a median *R*_0_*_c_*(*q_c_*) nearly equivalent to a predator-free system, the amplifying effect of chlorpyrifos, also acting through high toxicity to snail predators,^10,31–33^ was dampened owing to mild toxicity to snails^34^ and schistosome cercariae and miracidia.^35^ However, the net increase in transmission implies indirect top-down effects exerted greater influence on transmission than direct effects at environmental concentrations of chlorpyrifos.

Among the herbicides investigated across multiple pathways, atrazine increased transmission (*R*_0,atrazine_[EEC_atrazine_] 2·89, IQR 2·49–3·30) through indirect bottom-up stimulation of the snail carrying capacity even at concentrations well below peak EEC_atrazine_ (figure 3).^10,11^ Similar to chlorpyrifos, atrazine exhibited both direct and indirect effects on transmission. At low concentrations, which were more common in observations from the pesticide monitoring databases (appendix p 11), the net effect was dominated by bottom-up increases in transmission, but at higher concentrations, direct effects on schistosome larvae and snails reduced *R*_0,atrazine_ back towards baseline levels. Meanwhile glyphosate was estimated to eliminate transmission at environmental concentrations (*R*_0,glyphosate_[EEC_glyphosate_] 0· 00, IQR 0·00–0·00) owing to its high reproductive toxicity to intermediate host snails^36^ and its high environmental concentrations (figure 3).

**Figure 4:**
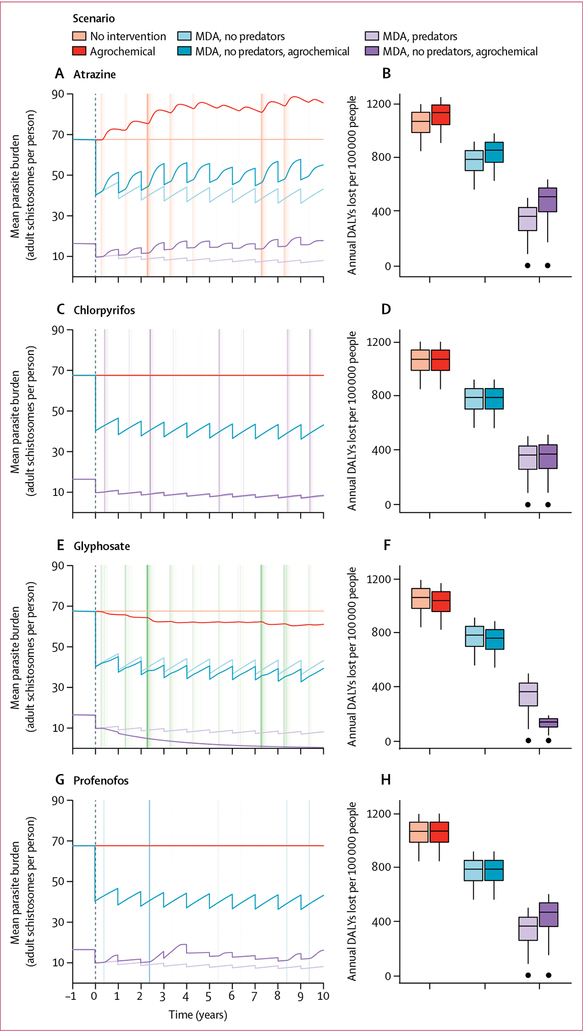
Effects of agrochemical pollution on human schistosomiasis control and disability Time series of mean parasite burden, expressed as adult schistosomes per person, estimated from dynamic model simulations across different intervention and agrochemical pollution scenarios (A, C, E, G), and distributions of cumulative DALYs lost in each scenario (B, D, F, H). Modelled agrochemical pollution scenarios for atrazine (A, B), chlorpyrifos (C, D), glyphosate (E,F), and profenofos (G, H) are shown. Shaded coloured backgrounds in each time series panel represent the concentration of each chemical through time as estimated from Pesticide in Water Calculator, with darker shading indicating concentrations closer to the peak EEC_c_. Darker lines and boxes in each panel represent scenarios with pollution of the indicated agrochemical and lighter lines indicate scenarios with no agrochemical effects. Across panels, red colours indicate scenarios with no intervention, blue colours represent annual mass drug administration interventions, and purple colours represent annual mass drug administration interventions and the presence or maintenance of a competent predator population that feeds on intermediate host snails. Malathion is not shown because it has no significant effect on transmission at environmentally relevant concentrations. DALY=disability-adjusted life-year. EEC_c_=expected environmental concentration. MDA=mass drug administration.

Atrazine was estimated to cause an additional 64·82 DALYs lost per 100,000 people per year (IQR 64·52 to 67·68) due to enhanced transmission of schistosomiasis over the course of an annual MDA campaign. Glyphosate might aid in reducing DALYs lost due to schistosomiasis during an annual MDA campaign with an estimated −22·26 DALYs lost per 100,000 people per year (−26·20 to −19·26). The insecticides chlorpyrifos, malathion, and profenofos did not have significant effects on transmission in MDA scenarios, but chlorpyrifos caused an additional 6·28 DALYs lost per 100,000 people per year (4·13 to 8·69) and profenofos an additional 103·06 DALYs lost per 100,000 people per year (89·63 to 104·90) in intervention scenarios that included a competent predator population that preys on intermediate host snails (figure 4; table 2).

Similar to the net effects of agrochemicals on *R0_c_*(*q_c_*), increases in transmission through time were largely driven by indirect effects on snail predators and algal dynamics. Short pulses of atrazine increase the snail population carrying capacity, leading to larger snail populations (appendix p 12) and quicker rebounds in infection following MDA (figure 4A). Similarly, temporary peaks in profenofos concentration cause high mortality rates in the snail predator population (appendix p 13), also leading to increased snail populations and higher rates of transmission to humans (figure 4D). Reductions in transmission due to glyphosate are caused by larger direct effects on snails that outweigh the bottom-up benefits conferred by glyphosate’s effects on algal dynamics (appendix p 14).

## Discussion

We developed a modelling framework evaluating the potential effects of agrochemicals on the transmission of *Schistosoma* trematodes using a large body of previous research and human survey data from ongoing epidemiological studies in the Senegal River basin. We found evidence that agrochemicals can affect the lifecycle of trematode parasites at environmentally relevant concentrations, but the consequences of these interactions for the transmission of schistosome species that affect more than 200 million people globally have only recently been considered.^4,10,11^ We estimated that agrochemical effects on schistosomiasis transmission caused up to 142·73 additional DALYs lost per 100,000 people per year in some scenarios. This disease burden was similar in magnitude to risks posed by a diet high in sodium, low physical activity, and lead exposure in Senegal in the 2017 Global Burden of Disease study.^37^ These risk factors are widely viewed as serious hazards to human health and are the target of policies and regulations seeking to reduce their effect. Agrochemical amplification of schistosomiasis transmission should be viewed similarly, and efforts to reduce disability associated with the interaction between agrochemicals and parasite transmission should be pursued, especially as agrochemical pollution extends into schistosomiasis-endemic areas. Our research can provide a basis for the design of follow-up experimental and observational research to further elucidate the human health effects of simultaneous chemical and biological exposures.

Alone the component effects of many agrochemicals, namely the herbicides atrazine, metolachlor, butachlor, butralin, pendimethalin, and glyphosate and the insecticides chlorpyrifos, profenofos, and endosulfan, on schistosome larvae, eggs, and snails would be expected to reduce schistosomiasis transmission, providing a modest protective effect. However, the lifespan of different stages of the schistosome lifecycle (eg, hours for miracidia and cercariae, weeks to months for sporocysts and snails, months to years for snail predators, and years for adult schistosomes) and the environmental persistence of different agrochemicals are highly variable. The net effects of agrochemicals through time is therefore determined by agrochemical persistence and by the stages of transmission affected. By investigating both the component and net effects of agrochemicals, we found that the net effect of chlorpyrifos, profenofos, and atrazine is to amplify transmission through the dominance of indirect effects on snail predators and algal dynamics, which are longer lasting than direct effects on snails and schistosome larvae.^10,38^ By contrast, snail predators appear to be more tolerant to the insecticide malathion, which has trivial net effects on transmission. Similarly, the widely used herbicide glyphosate decreases transmission owing to its reproductive toxicity to snails at environmentally relevant concentrations.^7^ Thus, there is substantial variability in agrochemical effects on transmission within the same type of agrochemical, suggesting that identification of agrochemical application regimens that retain productivity benefits without adversely affecting human health might be possible. However, these considerations should also be balanced with other effects of pesticide use on human health (eg, the carcinogenic effects of glyphosate).^39^

Indirect effects overwhelm and reverse the transmission-reducing, direct effects on snails and schistosome cercariae, miracidia, and eggs and might also be more likely to dominate in real-world settings, because they occur at lower concentrations that are more commonly observed (appendix p 11). The logarithmic response of snail populations to bottom-up stimulation of algal resources suggests that concentrations as low as a few parts per billion might be sufficient to alter the algal community in favour of the snail population. Previous mesocosm experiments corroborate this finding, showing enhanced snail populations in response to minimal nutrient increases.^10–12^ Multiple herbicides can affect algal community composition to benefit snail populations.^11,12,40,41^ Furthermore, eutrophication caused by nutrient-loaded agricultural runoff from fields using fertilisers is widespread and might become more common as fertiliser use increases.^42^ Eutrophication caused by increased human population densities, land use change, and dam infrastructure is associated with increased human exposure to trematode cercariae in the Mekong River basin, Chile, and around Lake Malawi.^43–45^ However, we were unable to derive dose-response functions for fertilisers owing to a lack of suitable dose-response data identified in the review. Further elucidation of the response between fertiliser-driven eutrophication and changes in schistosomiasis transmission represents a priority area for future research. Top-down effects driven by snail predator mortality can occur at low concentrations due to the high toxicity of insecticides to aquatic arthropods.^3,10^ Top-down effects of insecticides rely on the presence of a community similar to that characterised in the model in which arthropod snail predators are present, therefore we present results from models both with and without predators. Analyses suggest that predator populations, in particular *Macrobrachium* prawns, are established throughout coastal sub-Saharan Africa,^3^ and these same areas might be prone to insecticide contamination of surface waters.^46^ Ongoing efforts to establish either native or non-native prawn populations as an environmental control on schistosome-carrying snails^2,21,47^ will also need to consider the potential effects that low concentrations of insecticides might have on fragile introduced populations. Even in areas where arthropod predators of snails have a minimal role in the regulation of schistosomiasis transmission, agrochemicals might affect other species that feed on schistosome larvae or affect interspecies interactions, such as competition for resources.^48,49^

The realised effects of agrochemicals on schistosomiasis transmission will depend on local agricultural practices, crop types, application frequencies, rainfall and other environmental factors, species distributions, and human behaviours that affect exposure to schistosome parasites. Field measurements that quantify the fine-scale patterns of agrochemical pollution, snail and parasite densities, and their interaction will be essential to generate robust estimates of the effects of agrochemicals in particular settings. A prime example is an observational study in Kenya,^50^ which found that agrochemical pollution is a key determinant of local snail population density. Such data complements the agrochemical response functions fitted here, which characterise the fundamental biological responses of schistosome parasites, snail hosts, and snail predators to agrochemical exposures, and are therefore expected to be broadly applicable across diverse geographical landscapes.

All response functions were assumed to be monotonic across the range of considered concentrations and did not consider the possibility of threshold effects (eg, where excessive eutrophication of aquatic environments yields anoxic conditions that asphyxiate snails). Furthermore, experiments considering simultaneous agrochemical exposures were not identified; therefore, potential synergistic or antagonistic effects of multiple agrochemicals acting on the same pathway were not considered. For example, atrazine might reduce the activity of acetylcho linesterase in snails,^51^ which could further increase the toxicity of insecticides that also act through acetylcho linesterase inhibition, implying a potentially synergistic effect on snail mortality. Additionally, the reviewed literature described highly controlled experimental conditions, whereas exposure to agrochemicals in a natural setting would involve many simultaneous stressors, including predation, parasitism, and environmental fluctuations, possibly yielding different combined effects.

With respect to the mathematical model, derivation of *R*_0_(*q_c_*) required the conceptualisation of a steady-state population, which assumes a constant agrochemical effect even as agrochemical concentrations vary through time. Furthermore, we used a standard, but simple, representation of human infection, the negative binomially distributed community mean worm burden, to maintain analytical tractability for the *R*_0_(*q_c_*) analyses. Future research might benefit from implementation and simulation using stratified worm burden models^52^ to refine estimates of agrochemical impacts on transmission dynamics and disability. We present *R*_0_(*q_c_*) as a summary metric used to compare across parameters, agrochemicals, and types of effect that could potentially be used in further environmental risk assessment frameworks to determine regulatory limits informed by the potential for agrochemical effects on schistosomiasis transmission. We also explore the temporal domain of agrochemical effects to determine if the steady-state changes in *R*_0_(*q_c_*) translate to altered infection dynamics by incorporating agro chemical time series generated from the Pesticide in Water Calculator. These simulations show that agrochemical pollution might lead to increased rates of rebound in infection following MDA, perhaps necessitating greater MDA coverage or increased frequency to achieve the same reductions in disease burden as would be reached in an agrochemical-free setting.

Our findings motivate additional research on the potential effects of agrochemicals on schistosomiasis transmission. Increases in rural population density and the availability of modern agricultural inputs suggest that sub-Saharan Africa is on the verge of a rapid expansion of agrochemical use, suggesting that agrochemical pollution is likely to become more common in schistosomiasis-endemic areas.^6,53,54^ However, agrochemical data in these areas, including on quantities and types of chemicals being used, are sparse.^55^ Here, we raise concerns that the benefits generated by enhanced agricultural output with agrochemical use might be partially lost if they are accompanied by increased schistosomiasis transmission, especially considering the positive feedback loops that perpetuate poverty and tropical diseases.^56^

## Supplementary Material

SI

## Figures and Tables

**Table 1:** Studies identified in the systematic review

	Chemicals	Species	Transmission parameters investigated
Abdel-Ghaffar et al, 2016	Butralin, glyphosate, pendimethalin	*Schistosoma mansoni, Biomphalaria alexandrina*	Cercarial survival rate, miracidial survival rate, intermediate host reproduction rate, intermediate host mortality rate
Bajet et al, 2012	λ-cyhalothrin, deltamethrin, cypermethrin, chlorpyrifos, profenofos, malathion, carbaryl, 2,4-D, butachlor	*Macrobrachium lar*	Predator mortality rate
Bakry et al, 2011	Malathion, deltamethrin	*Helisoma duryi*	Intermediate host mortality rate
Bakry et al, 2012	Atrazine, glyphosate	*Biomphalaria alexandrina*	Intermediate host mortality rate
Bakry et al, 2016	Paraquat	*Lymnaea natalensis*	Intermediate host mortality rate
Barbieri et al, 2016	Carbofuran	*Macrobrachium olfersii*	Predator mortality rate
Baxter et al, 2011	Atrazine	*Physella spp*	Intermediate host carrying capacity
Benli et al, 2007	2,4-D	*Astacus leptodactylus*	Predator mortality rate
Bhavan et al, 2010	Carbaryl	*Macrobrachium malcomsonii*	Predator consumption rate
Browne and Moore, 2014	2,4-D	*Orconectes rusticus*	Predator consumption rate
Fornstrom et al, 1997	Terbufos	*Procambarus clarkii*	Predator mortality rate
Griggs et al, 2008	Atrazine, metolachlor	*Echinistoma trivolvis*	Cercarial survival rate
Gustafson et al, 2016	Atrazine	*Physa acuta*	Cercarial shedding rate
Halstead et al, 2015	Malathion, chlorpyrifos, terbufos, esfenvalerate, λ-cyhalothrin, permethrin	*Procambarus clarkii*	Predator mortality rate
Halstead et al, 2018	Atrazine, ammonium fertiliser, chlorpyrifos	*Bulinus truncatus, Schistosoma mansoni, Schistosoma haematobium, Procambarus alenii*	Intermediate host carrying capacity, intermediate host mortality rate, schistosome egg viability, cercarial survival rate, predator mortality rate
Hasheesh and Mohamed, 2011	Chlorpyrifos, profenofos	*Schistosoma haematobium, Bulinus truncatus*	Cercarial survival rate, miracidial survival rate, intermediate host mortality rate
Hussein et al, 2016	Other fertiliser	*Biomphalaria alexandrina*	Intermediate host mortality rate
Ibrahim et al, 1992	Chlorpyrifos	*Biomphalaria alexandrina*	Intermediate host mortality rate, intermediate host reproduction rate
Johnson et al, 2007	Ammonium fertiliser	*Planorbella trivolvis*	Intermediate host carrying capacity, intermediate host reproduction rate, cercarial shedding rate
Koprivnikar et al, 2006	Atrazine	*Echinistoma trivolvis*	Cercarial survival rate
Kristoff et al, 2011	Azinphos-methyl	*Biomphalaria glabrata*	Intermediate host reproduction rate
Lavarias et al, 2013	Fenitrothion	*Macrobrachium borellii*	Predator mortality rate
Leung et al, 1980	Paraquat	*Procambarus clarkia*	Predator mortality rate
Mohamed et al, 2012	Profenofos,diazinon	*Biomphalaria alexandrina*	Intermediate host mortality rate, intermediate host reproduction rate
Monde et al, 2016	Endosulfan	*Bulinus globusus*	Intermediate host mortality rate
Monte et al, 2016	Glyphosate	*Echinistoma paraensei*	Cercarial survival rate, miracidial survival rate
Naqvi et al, 1983	Trifluralin, oryzalin	*Procambarus clarkia*	Predator mortality rate
Naqvi et al, 1987	Endosulfan, trifluralin, monosodium methyl arsonate, Oust	*Procambarus clarkia*	Predator mortality rate
Oliveira et al, 2009	Endosulfan	*Biomphalaria tenagophila*	Intermediate host reproduction rate
Omkar and Rami, 1985	Endosulfan, phosphamidon, carbaryl	*Macrobrachium dayanum*	Predator mortality rate
Omkar and Shukla, 1984	Quinalphos, dichlorvos, monocrotophos, carbaryl	*Macrobrachium lamerii*	Predator mortality rate
Omran and Salama, 2013	Atrazine, glyphosate	*Biomphalaria alexandrina*	Intermediate host mortality rate
Ragab et al, 2006	Ammonium fertiliser, other fertiliser	*Biomphalaria alexandrina*	Intermediate host mortality rate
Revathi and Munuswamy, 2010	Tributyltin	*Macrobrachium rosenbergii*	Predator mortality rate
Rohr (unpublished data)	Malathion, chlorpyrifos, terbufos, esfenvalerate, λ-cyhalothrin, permethrin	*Macrobrachium rosenbergii*	Predator mortality rate
Rohr et al, 2008	Atrazine	*Planorbella trivolvis*	Intermediate host mortality rate, intermediate host carrying capacity
Rohr et al, 2008	Atrazine, carbaryl, malathion, glyphosate	*Echinistoma trivolvis*	Cercarial survival rate, intermediate host mortality rate, intermediate host reproduction rate
Sarojini et al, 1986	Fenitrothion	*Macrobrachium lamerii*	Predator mortality rate
Satapornvanit et al, 2009	Chlorpyrifos, dimethoate, profenofos	*Macrobrachium rosenbergii*	Predator mortality rate, predator consumption rate
Tantawy et al, 2002	Butachlor, fluazifop-p-butyl	*Biomphalaria alexandrina, Schistosoma mansoni*	Intermediate host mortality rate, cercarial survival rate, miracidial survival rate
Tchounwou et al, 1991	Malathion	*Bulinus havanensis, Planorbella trivolvis, Schistosoma mansoni*	Cercarial survival rate, miracidial survival rate, intermediate host mortality rate, intermediate host reproduction rate
Tchounwou et al, 1991	Ammonium fertiliser	*Schistosoma mansoni*	Miracidial survival rate, egg viability
Tchounwou et al, 1992	Malathion	*Schistosoma mansoni*	Cercarial survival rate

A version of this table with citations for included studies can be found in the appendix (pp 1–2).

**Table 2:** DALYs lost per 100 000 people per year attributable to each agrochemical in different intervention scenarios

	No intervention	Annual MDA	Annual MDA plus predators
Atrazine	58.62 (5443 to 64.58)	64.82 (62.52 to 67.68)	142.73 (122.00 to 144.95)
Chlorpyrifos	−0.45 (−1.55 to 1.37)	0.20 (−1.47 to 1.26)	6.28 (4.13 to 8.69)
Glyphosate	−24.76 (−26.32 to −22.33)	−22.26 (−26.20 to −19.26)	−212.47 (−243.04 to −100.91)
Malathion	−0.91 (−2.58 to 1.79)	0.19 (−1.09 to 1.14)	−0.39 (−1.00 to 0.26)
Profenofos	−0.16 (−1.74 to 1.81)	0.55 (−0.65 to 2.19)	103.06 (89.63 to 104.90)

Attributable DALYs are estimated as the difference between accumulated DALYs in agrochemically influenced and non-agrochemically influenced simulations. Shown are the median and IQR generated from 1000 simulations. DALYs=disability-adjusted life-years. MDA=mass drug administration.

## Research in context

### Evidence before this study

Previous investigation of the effect of agrochemical pollution on amphibian–trematode systems suggests that agrochemicals increase infection rates in amphibian hosts through increased exposure to infective trematode cercariae. Agrochemicals can have direct effects on trematode cercariae and miracidia and on intermediate snail hosts, and indirectly affect trematode lifecycles through e?ects on aquatic arthropods that are predators of snails and snail resources such as periphytic algae. These e?ects might also influence the transmission of human schistosomes. A mesocosm experiment investigating the e?ects of agrochemical additions on *Schistosoma* spp communities found that the introduction of insecticides, herbicides, and fertilisers all acted to benefit the intermediate host snail population and increase intermediate host infection with human schistosome species.

### Added value of this study

We systematically reviewed published literature estimating agrochemical effects on schistosome parasites, intermediate snail hosts, and snail predators and found evidence that agrochemicals probably affect schistosomiasis transmission through direct effects on cercariae, miracidia, and snails and through indirect effects on periphytic algae and snail predators that affect snail population dynamics. By use of dose-response functions fit to data extracted from the review, we estimated individual agrochemical effects on the agrochemically influenced basic reproduction number, and we simulated mass drug administration control efforts in the presence of agrochemicals. These simulations show that even short-term pulses of certain agrochemicals can alter transmission rates following control, potentially causing faster post-control rebounds in infection. Atrazine might cause the loss of additional disability-adjusted life-years (DALYs) through increases in schistosomiasis transmission driven by indirect effects that stimulate algal resources for intermediate host snails. In areas where aquatic arthropod predators suppress snail populations and schistosomiasis transmission, we also estimate that additional DALYs could be lost due to chlorpyrifos and profenofos pollution, both resulting from insecticide toxicity to aquatic arthropods and subsequent release of predation of the snail population. The insecticide malathion had minimal effects on schistosomiasis transmission dynamics and the herbicide glyphosate might reduce transmission through its high reproductive toxicity to snails.

### Implications of all the available evidence

Agricultural expansion has emerged as a prominent driver of environmental change in the 21st century and understanding its effects on human health is essential. Agrochemical pollution has been implicated in amphibian population declines due in part to its effects on trematode parasites that infect amphibians. The same pathways that affect transmission of these parasites probably affect the transmission of the human parasites that cause schistosomiasis, a disease that affects at least 200 million people globally. Agrochemicals have the potential to increase schistosomiasis transmission at environmentally relevant concentrations, which might interrupt ongoing efforts to control and eliminate the disease in many areas where it is endemic. Variability in the potential for di?erent agrochemicals of the same type (eg, herbicide and insecticide) might provide an opportunity to mitigate the transmission-increasing effects of agrochemicals while maintaining their benefit to agriculture.
