# Supplementary material for: Effects of agrochemical pollution on schistosomiasis transmission: a systematic review and modelling analysis
Source: Lancet Planet Health. Author manuscript; Available in PMC 2020 Dec 22. (PMC7754781; doi:10.1016/S2542-5196(20)30105-4)
Supplement: SI [file NIHMS1646800-supplement-SI.pdf]

THE LANCET
Planetary Health

Supplementary appendix

This appendix formed part of the original submission and has been peer reviewed. We post it as supplied by the authors.

Supplement to: Hoover CM, Rumschlag SL, Strgar L, et al. Effects of agrochemical pollution on schistosomiasis transmission: a systematic review and modelling analysis. *Lancet Planet Health* 2020; **4:** e280–91.

Supplementary Information

Systematic Review Summaries

Table S1: Studies identified in the systematic review, along with study citations, chemicals, species, and parameters investigated.

Reference	Chemical(s)	Species	Transmission parameter(s) investigated
**Abdel-Ghaffar et al 2016**[1]	Butralin, Glyphosate, Pendimethalin	*Schistosoma mansoni, Biomphalaria alexandrina*	Cercarial survival rate, miracidial survival rate, intermediate host reproduction rate, intermediate host mortality rate
**Bajet et al 2012**[2]	Lambda-cyhalothrin, Deltamethrin, Cypermethrin, Chlorpyrifos, Profenofos, Malathion, Carbaryl, 2,4-D, Butachlor	*Macrobrachium lar*	Predator mortality rate
**Bakry et al 2011**[3]	Malathion, Deltamethrin	*Helisoma duryi*	Intermediate host mortality rate
**Bakry et al 2012**[4]	Atrazine, Glyphosate	*Biomphalaria alexandrina*	Intermediate host mortality rate
**Bakry et al 2016**[5]	Paraquat	*Lymnaea natalensis*	Intermediate host mortality rate
**Barbieri et al 2016**[6]	Carbofuran	*Macrobrachium olfersii*	Predator mortality rate
**Baxter et al 2011**[7,8]	Atrazine	*Physella spp*	Intermediate host carrying capacity
**Benli et al 2007**[9]	2,4-D	*Astacus leptodactylus*	Predator mortality rate
**Bhavan et al 2010**[10]	Carbaryl	*Macrobrachium malcomsonii*	Predator consumption rate
**Browne & Moore 2014**[11]	2,4-D	*Orconectes rusticus*	Predator consumption rate
**Fornstrom et al 1997**[12]	Terbufos	*Procambarus clarkii*	Predator mortality rate
**Griggs et al 2008**[13]	Atrazine, Metolachlor	*Echinistoma trivolvis*	Cercarial survival rate
**Gustafson et al 2016**[14]	Atrazine	*Physa acuta*	Cercarial shedding rate
**Halstead et al 2015**[15]	Malathion, Chlorpyrifos, Terbufos, Esfenvalerate, Lambda-cyhalothrin, Permethrin	*Procambarus clarkii*	Predator mortality rate
**Halstead et al 2018**[16]	Atrazine, Ammonium Fertilizer, Chlorpyrifos	*Bulinus truncatus, Schistosoma mansoni, Schistosoma haematobium, Procambarus alenii*	Intermediate host carrying capacity, intermediate host mortality rate, schistosome egg viability, cercarial survival rate, predator mortality rate
**Hasheesh & Mohamed 2011**[17]	Chlorpyrifos, Profenofos	*Schistosoma haematobium, Bulinus truncatus*	Cercarial survival rate, miracidial survival rate, intermediate host mortality rate
**Hussein et al 2016**[18]	Other Fertilizer	*Biomphalaria alexandrina*	Intermediate host mortality rate
**Ibrahim et al 1992**[19]	Chlorpyrifos	*Biomphalaria alexandrina*	Intermediate host mortality rate, intermediate host reproduction rate
**Johnson et al 2007**[20]	Ammonium Fertilizer	*Planorbella trivolvis*	Intermediate host carrying capacity, intermediate host reproduction rate, cercarial shedding rate
**Koprivnikar et al 2006**[21]	Atrazine	*Echinistoma trivolvis*	Cercarial survival rate
**Kristoff et al 2011**[22]	Azinphos-methyl	*Biomphalaria glabrata*	Intermediate host reproduction rate
**Lavarias et al 2013**[23]	Fenitrothion	*Macrobrachium borellii*	Predator mortality rate
**Leung et al 1980**[24]	Paraquat	*Procambarus clarkii*	Predator mortality rate

**Mohamed et al 2012**[25]	Profenofos,Diazinon	*Biomphalaria alexandrina*	Intermediate host mortality rate, intermediate host reproduction rate
**Monde et al 2016**[26]	Endosulfan	*Bulinus globusus*	Intermediate host mortality rate
**Monte et al 2016**[27]	Glyphosate	*Echinistoma paraensei*	Cercarial survival rate, miracidial survival rate
**Naqvi et al 1983**[28]	Trifluralin, Oryzalin	*Procambarus clarkii*	Predator mortality rate
**Naqvi et al 1987**[29]	Endosulfan, Trifluralin, MSMA, Oust	*Procambarus clarkii*	Predator mortality rate
**Oliveira et al 2009**[30]	Endosulfan	*Biomphalaria tenagophila*	Intermediate host reproduction rate
**Omkar & Rami 1985**[31]	Endosulfan, Phosphamidon, Carbaryl	*Macrobrachium dayanum*	Predator mortality rate
**Omkar & Shukla 1984**[32]	Quinalphos, Dichlorvos, Monocrotophos, Carbaryl	*Macrobrachium lamerii*	Predator mortality rate
**Omran & Salama 2013**[33]	Atrazine, Glyphosate	*Biomphalaria alexandrina*	Intermediate host mortality rate
**Ragab et al 2006**[34]	Ammonium Fertilizer, Other Fertilizer	*Biomphalaria alexandrina*	Intermediate host mortality rate
**Revathi & Munuswamy 2010**[35]	Tributyltin	*Macrobrachium rosenbergii*	Predator mortality rate
**Rohr (unpublished data)**	Malathion, Chlorpyrifos, Terbufos, Esfenvalerate, Lambda-cyhalothrin, Permethrin	*Macrobrachium rosenbergii*	Predator mortality rate
**Rohr et al 2008**[36]	Atrazine	*Planorbella trivolvis*	Intermediate host mortality rate, intermediate host carrying capacity
**Rohr et al 2008b**[37]	Atrazine, Carbaryl, Malathion, Glyphosate	*Echinistoma trivolvis*	Cercarial survival rate, intermediate host mortality rate, intermediate hose reproduction rate
**Sarojini et al 1986**[38]	Fenitrothion	*Macrobrachium lamerii*	Predator mortality rate
**Satapornvanit et al 2009**[39]	Chlorpyrifos, Dimethoate, Profenofos	*Macrobrachium rosenbergii*	Predator mortality rate, predator consumption rate
**Tantawy et al 2002**[40]	Butachlor, Fluazifop-p-butyl	*Biomphalaria alexandrina, Schistosoma mansoni*	Intermediate host mortality rate, cercarial survival rate, miracidial survival rate
**Tchounwou et al 1991**[41]	Malathion	*Bulinus havanensis, Planorbella trivolvis, Schistosoma mansoni*	Cercarial survival rate, miracidial survival rate, intermediate host mortality rate, intermediate host reproduction rate
**Tchounwou et al 1991b**[42]	Ammonium Fertilizer	*Schistosoma mansoni*	Miracidial survival rate, egg viability
**Tchounwou et al 1992**[43]	Malathion	*Schistosoma mansoni*	Cercarial survival rate

Table S2: Number of fitted dose-response functions from the systematic review across agrochemical type and type of effect

	**Bottom-up**	**Top-down**	**Direct Snail**	**Direct Schistosome**
**Fertilizers**	0	0	6	6
**Herbicides**	1	9	14	18
**Insecticides**	0	41	17	8

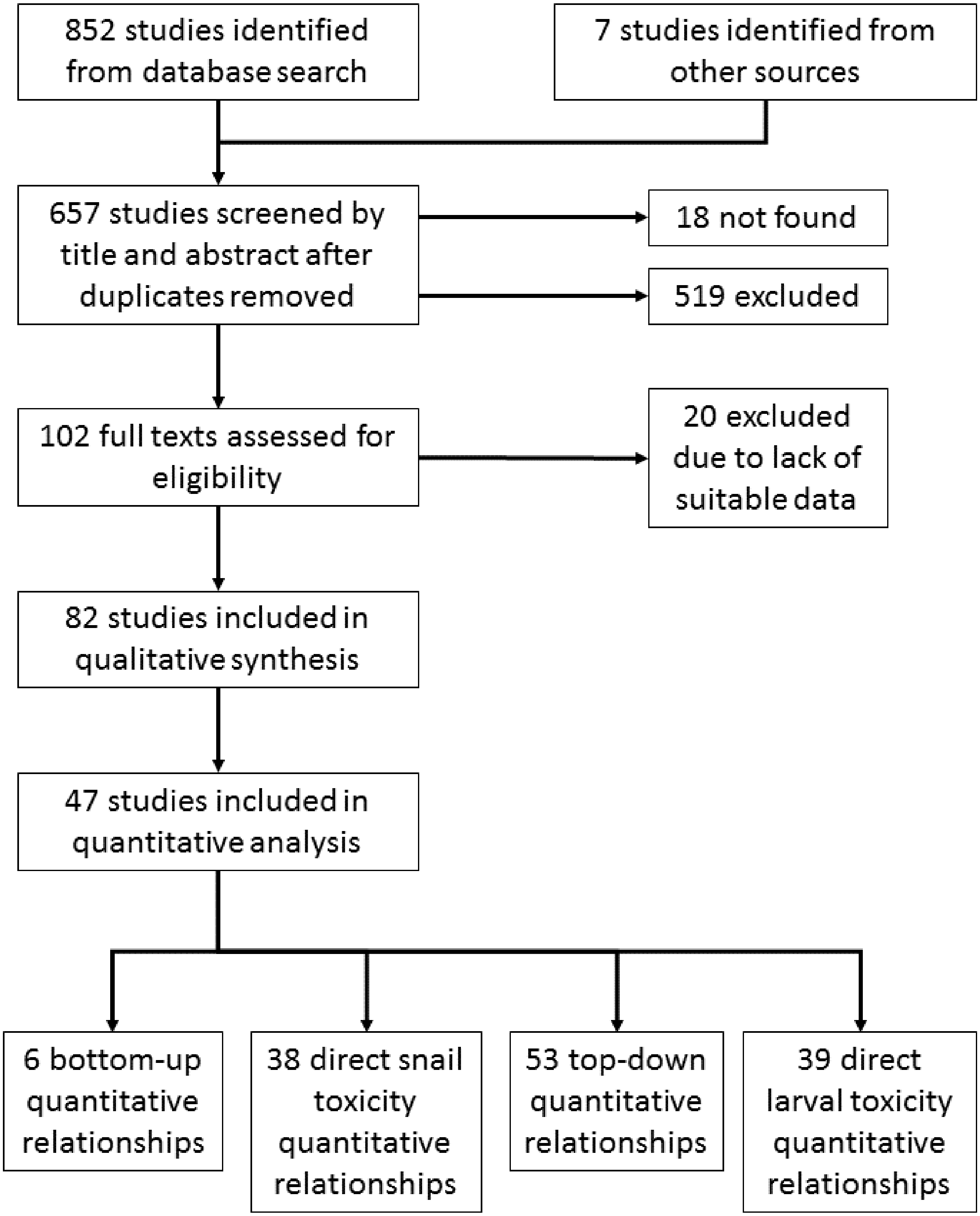

Figure S1: Systematic review PRSMA summary with final outcomes across pathways of effect.

Generation of Estimated Environmental Concentrations and Peak Observed Concentrations

We focused on the 35 unique herbicides and insecticides identified in the literature review. Because fertilizers were not well studied in the literature and they are designed to break down into essential nutrients, they were not considered here. For 9 herbicides and 12 insecticides, we were able to identify chemical properties and application recommendations listed in the supplementary spreadsheets. These agrochemicals are commonly applied through much of the world, while the remaining 14 pesticides have mostly been banned. We generated estimated environmental concentrations (EECs) using the Pesticide in Water Calculator v 1.52 (PWC).[44] The PWC is a graphic user interface developed by the EPA Office of Pesticide Programs. The PWC links the output of the Pesticide Root Zone Model (PRZM) and the Variable Volume Water Model (VVWM). We used the PWC to generate EECs in a typical scenario used in environmental risk assessments: a fixed volume, no flow, 5.26-ha by 2.74-m reservoir receiving runoff and overspray from an adjacent 10-ha pesticide-treated field.[44–46] Using mass balance equations, the PWC assumes that all materials in the water and sediment, two compartments, are at thermodynamic equilibrium. Pesticides are delivered via the inflow of dissolved pesticides or through drift to the water. Sorbed pesticides are delivered to the water and sediment. Pesticides leave the system through sediment burial, volatilization, and degradation.

Data describing the environmental mobility and persistence of pesticides used as model inputs for the generation of EECs by the PWC were gathered from sources as indicated in the supplementary spreadsheets. Model inputs for data concerning the number of applications per year, timing of applications, and maximum recommended application rates and methods of application were taken from commercial pesticide product labels (see supplementary spreadsheets). For all pesticides, water, benthic, and soil reference temperatures were assumed to be 23°C, and photolysis reference latitude was 40°. When foliar half-life was not available for a given pesticide, foliar half-life was assumed not to be a contributor to environmental breakdown and was set to zero. According to the PWC user manual, efficiency was 0.99 and drift was 0.01. Applications were assumed to occur every year. For each pesticide compound EECs were generated for reservoirs in MS as the geographic and climatic variability here was estimated to be most similar to areas of concern in SSA.

Three of the most comprehensive pesticide monitoring databases in the United States, the U.S. Geological Service (USGS) National Water Quality Assessment (NAWQA), the 2013 USGS Midwest Stream Quality Assessment (MSQA) and the California Department of Pesticide Regulation Surface Water Database (CA-SURF), were used to determine peak observed concentrations. Figure S2 below shows the distribution of concentrations above the detection limit from each database for the agrochemicals explored in depth (those with sufficient dose-response functions derived from the review). The peak observed concentration for each chemical (POC_c) was simply the maximum observed concentration across the three databases.

Dose-response function fitting

Logistic-shaped functions typical of dose-response data were fit to raw data using the R package *drc* [47] or regenerated from reported parameters. Two exceptions to this general procedure—snail carrying capacity response to herbicide concentration and agrochemical toxicity to schistosome miracidia and cercariae—are explained further below. To relate agrochemical responses between different studies and agrochemicals, functions estimate the proportional change in the baseline parameter value, P, listed in Table S1.

Toxicity to schistosome larvae at a daily timescale

To model agrochemical toxicity to schistosome larvae, whose lifespan is on the order of hours rather than days, at the same daily timescale as the rest of the model, we estimate the mean number of hours of survival of each larval stage under different agrochemical scenarios. Data from study j investigating agrochemical c's toxicity to larvae are reported as time series of larval survival at different agrochemical concentrations over the course of a day or several hours. We first fit a general logistic curve to each of these time series to derive $LC_{50,jc}$ and slp_{jc} parameters describing the survival of larvae in the experiment over. We next fit simple log-linear or linear models of $LC_{50,jc}$ and slp_{jc} with respect to agrochemical concentration, q. We thus arrive at a function to estimate a time series of larval survival, π, given agrochemical concentration, q, across time, t, in hours. Integrating this function from $t = 0 - 24\ hrs$ at q (eqn 17) provides an estimate of the expected number of miracidia- or cercariae-hours of survival in a

given day with agrochemical concentration, q. The function estimating study-and agrochemical-specific relative changes in larval survival to agrochemical concentration is then derived via comparison to the study control group according to equation 18 below.

$$\boldsymbol{\pi_{jc}(t,q)} = \int_0^{24} \frac{\mathbf{1}}{\mathbf{1} + \mathbf{exp}\left(\boldsymbol{slp_{jc}(q)\,log}\left(\frac{\boldsymbol{t}}{\boldsymbol{LC_{50,jc}(q)}}\right)\right)}\boldsymbol{dt} \tag{17}$$

$$\boldsymbol{f_{jc}(q)} = \frac{\boldsymbol{\pi_{jc}(q)}}{\boldsymbol{\pi_{jc}(0)}} \tag{18}$$

Mathematical model

Snails

The model described here builds on our previous work [16,48] and on classical models of schistosomiasis transmission [49,50] to incorporate a variety of agrochemical effects that may affect transmission. Intermediate host snails follow an S-E-I framework in which new snails enter the system at agrochemically-sensitive daily fertility rate, f_N(q). This fertility rate is moderated by density dependence in the form of a classic logistic growth model in which the carrying capacity, $\Phi_N(q)$, changes in response to agrochemical inputs. Susceptible and exposed snails contribute to reproduction, while infected snails are assumed to be castrated by parasitic infection.[51] Susceptible snails become exposed, E, following contact with miracidia, M, at a per miracidia-hour of exposure probability, β. This probability roughly translates to the person-to-snail transmission probability and was estimated by fitting our previous model to epidemiological data. [16] The parameter Ω represents the proportion of overlap between area where miracidia hatch and where susceptible snails reside, but is set to unity currently. Exposed snails next become infected, I, following a latent period, σ, that corresponds to sporocyst development in exposed snails. Snails in each class die at a background daily mortality rate, $\mu_N(q)$, that can increase due to agrochemical toxicity. Infected snails are subject to additional infection-induced mortality at rate, μ_I. Finally, snails of each class can be consumed by the predator population, P, at a rate, ψ, determined by the predator attack rate, handling time, and snail density (details below).

Predator population

New predators enter the system at daily fertility rate, f_P, which is also moderated by density dependence in the form of a logistic growth equation with carrying capacity, K_P. Predators also die at daily rate, $\mu_P(q)$, which is increased according to insecticide concentration. Predators attack the different infection classes of the snail population based on the daily, per-predator consumption rate, $\psi(q)$, which may decrease at sub-lethal concentrations of insecticide concentration according to the function $f_{jc}(q)$. The consumption rate is a function of the attack rate, α, the handling time, T_h, the density of the snail infection class, and an exponent on the density, n, which regulates the shape of the response function relating the consumption rate to snail density. We set n to unity here to generate a logistic shape function commonly referred to as Holling's disk equation [52] in which the consumption rate rises steadily before reaching an asymptote that is roughly equivalent to T_h^{-1}, the maximum number of snails consumed by a given predator in a day. We do not explicitly tie predator population dynamics to snail consumption as the arthropod predators under consideration are generalists and are therefore assumed to readily switch to other food sources when snails are not present.

Human infection

Human infections are followed as the mean worm burden, W, in the human population, H. New adult worms arise from cercariae, C, that are shed from infected snails at daily rate, θ, and undergo the process of locating a human host, successfully infecting that host, and maturing into an adult worm with hourly probability, λ. This parameter roughly translates to the snail-to-human transmission probability and was also estimated via fit to epidemiological data (described below). Adult worms die at daily rate, μ_W, and human hosts harboring adult worms die at daily rate μ_H. Since only mated adult female worms, W_f contribute to transmission, their population is estimated separately assuming a 1:1 sex ratio and mating probability, $\gamma(W,\kappa)$, as a function of the mean worm burden, W, and the dispersal of adult worms among the human population assuming a negative binomial distribution, κ. [49,53]

Schistosome larvae

To incorporate sub-daily dynamics of agrochemical toxicity on schistosome larvae, we estimate the number of potential infectious miracidia-hrs, $M(t)$, and cercariae-hrs, $C(t)$, in a given 24-hr day as described above. Schistosome eggs are laid by adult female worms at daily rate, m, and hatch in proportion to agrochemical-sensitive viability, $v(q)$. The parameter $\pi_M(q)$ then represents the mean number of hours a miracidia that has successfully hatched is expected to be infectious, given agrochemical concentration, q. Similarly, cercariae shed by infected snails, I, at daily, agrochemical-dependent rate, $\theta(q)$, are infectious for mean $\pi_C(q)$ hours. The procedure to estimate response functions that determine $\pi_M(q)$ and $\pi_C(q)$ at variable concentrations of different agrochemicals are described further above.

$$dS/dt = f_N(q)\left(1 - \frac{N}{K_N(q)}\right)(S+E) - \mu_N(q)S - P\psi(q)S^n - \beta\Omega MS \quad (1)$$

$$dE/dt = \beta\Omega MS - \mu_N(q)E - P\psi(q)E^n - \sigma E \quad (2)$$

$$dI/dt = \sigma E - (\mu_N(q) + \mu_I)I - P\psi(q)I^n \quad (3)$$

$$dW/dt = \lambda\Omega C - (\mu_H + \mu_W)W \quad (4)$$

$$dP/dt = f_P\left(1 - \frac{P}{K_P}\right)P - \mu_P(q)P \quad (5)$$

$$M(t) = W_F m v(q)\pi_M(q) \quad (6)$$

$$W_F = \frac{WH\gamma(W,k)}{2} \quad (7)$$

$$C(t) = I\theta(q)\pi_C(q) \quad (8)$$

$$\psi(q) = \frac{\alpha f_{jc}(q)}{1 + \alpha T_h N^n} \quad (9)$$

$$N = S + E + I \quad (10)$$

Table S4. Parameter definitions and values used in the model and $R_0(q)$ expression

Symbol	Description	Value	Source
**Constant Parameters**			
$\boldsymbol{\beta}$	Infection probability from person-to-snail; exposed snails per miracidia-hour of exposure per day	$6.94\text{x}10^{-7}$	Fit to epi data
$\boldsymbol{\lambda}$	Infection probability from snail-to-human; adult worms established in human hosts per cercariae-hour of exposure per day	$4.08\text{x}10^{-6}$	Fit to epi data
$\boldsymbol{\sigma}$	Daily rate of schistosomulae development; infected snails per exposed snail per day	0.025	[49]
$\boldsymbol{\Omega}$	Degree of overlap between water contamination, snail, and human habitats	1	Site-specific
$\boldsymbol{\mu_I}$	Increased daily mortality rate of infected snails due to parasite infection	0.083	[49]
$\boldsymbol{f_P}$	Per-capita daily fertility rate of predator population including survival to effective snail predation	0.82	[54]
$\boldsymbol{K_P}$	Carrying capacity (Pm^{-2})of predator population	0.125	
n	Exponent of prey density in Holling functional response	1	[55]
$\boldsymbol{T_h}$	Handling time from Holling functional response; approximately the inverse of the daily maximum snails consumed per predator	0.067	[56]
$\boldsymbol{\kappa}$	Clumping parameter of the negative binomial distribution of worms within the human population	0.08	Estimated from epi data
H	Human density (Hm^{-2}) utilizing water contact site	1.5	[48]
$\boldsymbol{\mu_W}$	Natural per-capita daily mortality rate of adult worms (assuming mean lifespan of 3.3 years)	$8.3\text{x}10^{-4}$	[57]
$\boldsymbol{\mu_H}$	Per-capita daily mortality rate of adult worms caused by human mortality (assuming mean lifespan of 60 years)	$4.57\text{x}10^{-5}$	[48]
**Agrochemical sensitive parameters**			
$\boldsymbol{K_N(q)}$	Snail carrying capacity (Nm^{-2}) influenced by agrochemicals through bottom-up stimulation of algal resources	50	[58]
$\boldsymbol{f_N(q)}$	Per-capita daily fertility rate of snails including survival to detectability	0.60	[58]
$\boldsymbol{\mu_N(q)}$	Per-capita daily snail mortality rate influenced by direct toxicity of agrochemicals	0.017	[58]
$\boldsymbol{v(q)}$	Schistosome egg viability: daily number of miracidia per schistosome egg, influenced by direct toxicity of agrochemicals	0.084	[16]
$\boldsymbol{\pi_M(q)}$	Mean number of miracidia-hours per day	6.22	[41]
$\boldsymbol{\theta(q)}$	Shedding rate of infected snails (I): cercariae per infected snail per day, influenced by bottom up stimulation of algal resources	109	[59]
$\boldsymbol{\pi_C(q)}$	Mean number of cercariae-hours per day	14.21	[43]
$\boldsymbol{\alpha(q)}$	Per capita attack rate of predators on snails influenced by sub-lethal agrochemical toxicity to predators	0.04	[60]
$\boldsymbol{\mu_P(q)}$	Per-capita daily mortality rate of predators influenced by lethal agrochemical toxicity	0.038	[16]

Model fit

An agrochemical-free version of the model was previously fit to epidemiological data from a school-based mass drug administration campaign in a community near Saint Louis, Senegal. [16,48,61] Briefly, the mean worm burden, state variable W from the model, is simulated through time with different candidate transmission parameters, β and λ. The R function *optim* is used to estimate the best fit parameters via maximum likelihood. To estimate parametric

uncertainty associated with model fit, a profile likelihood approach is next used to determine the 95% confidence interval of the transmission parameters. Uncertainty in the transmission parameters is then incorporated into Monte Carlo simulations by weighting parameter sets by the inverse of their negative log likelihood.

MDA Implementation and DALYs Estimation

Mass drug administration in the model is implemented by dividing the mean worm burden compartment into treated, W_T, and untreated, W_U, compartments according to MDA coverage, $\mathcal{C}$, such that population mean worm burden, $W = \mathcal{C}W_T + (1-\mathcal{C})W_U$. When implementing MDA, W_T is instantaneously reduced according to MDA efficacy, $\mathcal{E}$ such that $W_T^{MDA} = W_T(1-\mathcal{E})$. We assume the negative binomial dispersion parameter, κ, is constant through time between the two populations.

Disability weights associated with schistosomiasis infection are determined by whether an individual has a heavy (defined by the WHO for *S. haematobium* as ≥ 50 eggs/10mL urine) or light infection (defined by the WHO as > 0, but < 50 eggs/10mL urine), therefore DALYs estimation requires estimates of the number of individuals with heavy and light infections, H_{hi} and H_{lo}, respectively. To convert model mean worm burden values to estimates of H_{hi} and H_{lo}, we first sample $n_T = H\mathcal{C}$ draws from a negative binomial distribution with mean W_T and dispersion κ to sample individual worm burdens among the treated segment of the human population. Similarly, we sample $n_U = H(1-\mathcal{C})$ draws from a negative binomial distribution with mean W_U and dispersion κ for the untreated segment of the human population. These individual worm burden estimates, denoted W_h, are then converted to individual egg burden estimates, denoted B_h, as: $B_h = 0.5W_h\gamma(W_h)\epsilon$, where $0.5W_h\gamma(W_h)$ represents an estimate of the number of mated (i.e. egg-producing) female worms, and ϵ is the estimated number of eggs per mated female worm per 10mL urine from [62]. With the full distribution of B_h, we then estimate H_{hi} as the number of individuals with $B_h \geq 50$ and H_{lo} as the number of individuals with $0 < B_h < 50$. Next, we incorporate disability weights associated with heavy and light infection (DW_{hi} and DW_{lo}, respectively) normalized to daily values to match the time step of the model to estimate accumulated disability over a simulation period of T_{sim} days:

$$\boldsymbol{DALYs = \sum_{t=1}^{T_{sim}} \frac{DW_{hi}}{365} H_{hi_t} + \frac{DW_{lo}}{365} H_{lo_t}} \quad \textbf{(11)}$$

Cumulative DALYs estimates are then normalized to DALYs per 100,000 population per year for comparison to other risk factors.

Table S5. Parameter definitions and values used in MDA implemenatation and DALYs estimation

Symbol	Description	Value	Source
**MDA and DALYs Parameters**			
$\boldsymbol{\mathcal{C}}$	MDA coverage of human population	0.8	
$\boldsymbol{\mathcal{E}}$	MDA efficacy	0.93	
$\boldsymbol{\epsilon}$	Eggs produced per mated female worm per day per 10mL urine	**5**.2	[62]
$\boldsymbol{DW_{hi}}$	Disability weight associated with heavy infection intensity	0.05	[63–65]
$\boldsymbol{DW_{hi}}$	Disability weight associated with light infection intensity	0.014	[63–65]

R_0(q) derivation

We employ the next generation matrix methodology to develop an analytic expression for *$R_0(q)$*, the agrochemical-sensitive basic reproduction number. We define *$R_0(q)$* as the expected number of mated female worms produced by a single mated female worm in an entirely susceptible human host population within an ecosystem where agrochemical concentration is equal to *q*. We begin by linearizing the disease system about the disease free steady state by solving eqn 11 and eqn 12 for the equilibrium number of snails, N*, and predators, P*, respectively.

$$\boldsymbol{0 = f_N(q)\left(1 - \frac{N^*}{K_N(q)}\right) - \mu_N(q) - P^*\psi^*(q)(N^*)^n} \quad (12)$$

$$\mathbf{0} = \boldsymbol{f_P}\left(\mathbf{1} - \frac{\boldsymbol{P^*}}{\boldsymbol{K_P}}\right) - \boldsymbol{\mu_P(q)} \tag{13}$$

Substituting expressions for N* and P*, we subsequently restrict our attention to the linearized subsystem of equations governing infectious state dynamics (eqn 13 – 15).

$$\frac{\boldsymbol{dE}}{\boldsymbol{dt}} = \boldsymbol{\beta\Omega M N^*} - \left(\boldsymbol{\mu_N(q)} + \boldsymbol{P^*\psi^*(q)} + \boldsymbol{\sigma}\right)\boldsymbol{E} \tag{14}$$

$$\frac{\boldsymbol{dI}}{\boldsymbol{dt}} = \boldsymbol{\sigma E} - \left(\boldsymbol{\mu_N(q)} + \boldsymbol{\mu_I} + \boldsymbol{P^*\psi^*(q)}\right)\boldsymbol{I} \tag{15}$$

$$\frac{\boldsymbol{dW}}{\boldsymbol{dt}} = \boldsymbol{\lambda\Omega C} - \left(\boldsymbol{\mu_H} + \boldsymbol{\mu_W}\right)\boldsymbol{W} \tag{16}$$

Following the next generation matrix method we build matrices T and Σ corresponding to the generation of new infections and transitions between infected states, respectively, and we take the product T(-Σ$^-$) to produce the system's next generation matrix with large domain, K_L.

$$T = \begin{pmatrix} 0 & 0 & \beta\Omega\left(\frac{\mathrm{Hm\upsilon(q)\pi_M}(q)}{2}\right)N^* \\ 0 & 0 & 0 \\ 0 & \lambda\Omega\Theta(\mathrm{q})\pi_{\mathrm{C}}(q) & 0 \end{pmatrix}$$

$$-\Sigma^- = \begin{pmatrix} \frac{1}{\mu_N(q) + P^*\psi^*(q) + \sigma} & 0 & 0 \\ \frac{\sigma}{(\mu_N(q) + P^*\psi^*(q) + \sigma)(\mu_N(q) + \mu_I + P^*\psi^*(q))} & \frac{1}{\mu_N(q) + \mu_I + P^*\psi^*(q)} & 0 \\ 0 & 0 & \frac{1}{\mu_H + \mu_W} \end{pmatrix}$$

$$K_L = \begin{pmatrix} 0 & 0 & \frac{\beta\Omega\mathrm{Hm\upsilon(q)\pi_M}(q)N^*}{2(\mu_H + \mu_W)} \\ 0 & 0 & 0 \\ \frac{\sigma\lambda\Omega\Theta(\mathrm{q})\pi_{\mathrm{C}}(q)}{(\mu_N(q) + P^*\psi^*(q) + \sigma)(\mu_N(q) + \mu_I + P^*\psi^*(q))} & \frac{\lambda\Omega\Theta(\mathrm{q})\pi_{\mathrm{C}}(q)}{\mu_N(q) + \mu_I + P^*\psi^*(q)} & 0 \end{pmatrix}$$

K_L reduces to K (shown below) the next generation matrix for only those states which are states at infections, E and W.

$$K = \begin{pmatrix} 0 & \frac{\beta\Omega\mathrm{Hm\upsilon(q)\pi_M}(q)N^*}{2(\mu_H + \mu_W)} \\ \frac{\sigma\lambda\Omega\Theta(\mathrm{q})\pi_{\mathrm{C}}(q)}{(\mu_N(q) + P^*\psi^*(q) + \sigma)(\mu_N(q) + \mu_I + P^*\psi^*(q))} & 0 \end{pmatrix}$$

Given K, we can compute the spectral radius (largest eigen value), which is interpreted as $R_0(q)$. Thus, $R_0(q)$ is given by:

$$\boldsymbol{R_0(q)} = \sqrt{\frac{\left(\boldsymbol{\sigma\lambda}\boldsymbol{\Omega\theta}(\mathbf{q})\boldsymbol{\pi}_{\mathbf{C}}(\boldsymbol{q})\right)(\boldsymbol{\beta}\boldsymbol{\Omega}\mathbf{Hm}\boldsymbol{\upsilon}(\mathbf{q})\boldsymbol{\pi}_{\mathbf{M}}(\boldsymbol{q})\boldsymbol{N}^{*})}{\mathbf{2}(\boldsymbol{\mu_N}(\boldsymbol{q}) + \boldsymbol{P}^{*}\boldsymbol{\psi}^{*}(\boldsymbol{q}) + \boldsymbol{\sigma})(\boldsymbol{\mu_N}(\boldsymbol{q}) + \boldsymbol{\mu_I} + \boldsymbol{P}^{*}\boldsymbol{\psi}^{*}(\boldsymbol{q}))(\boldsymbol{\mu_H} + \boldsymbol{\mu_W})}} \quad (17)$$

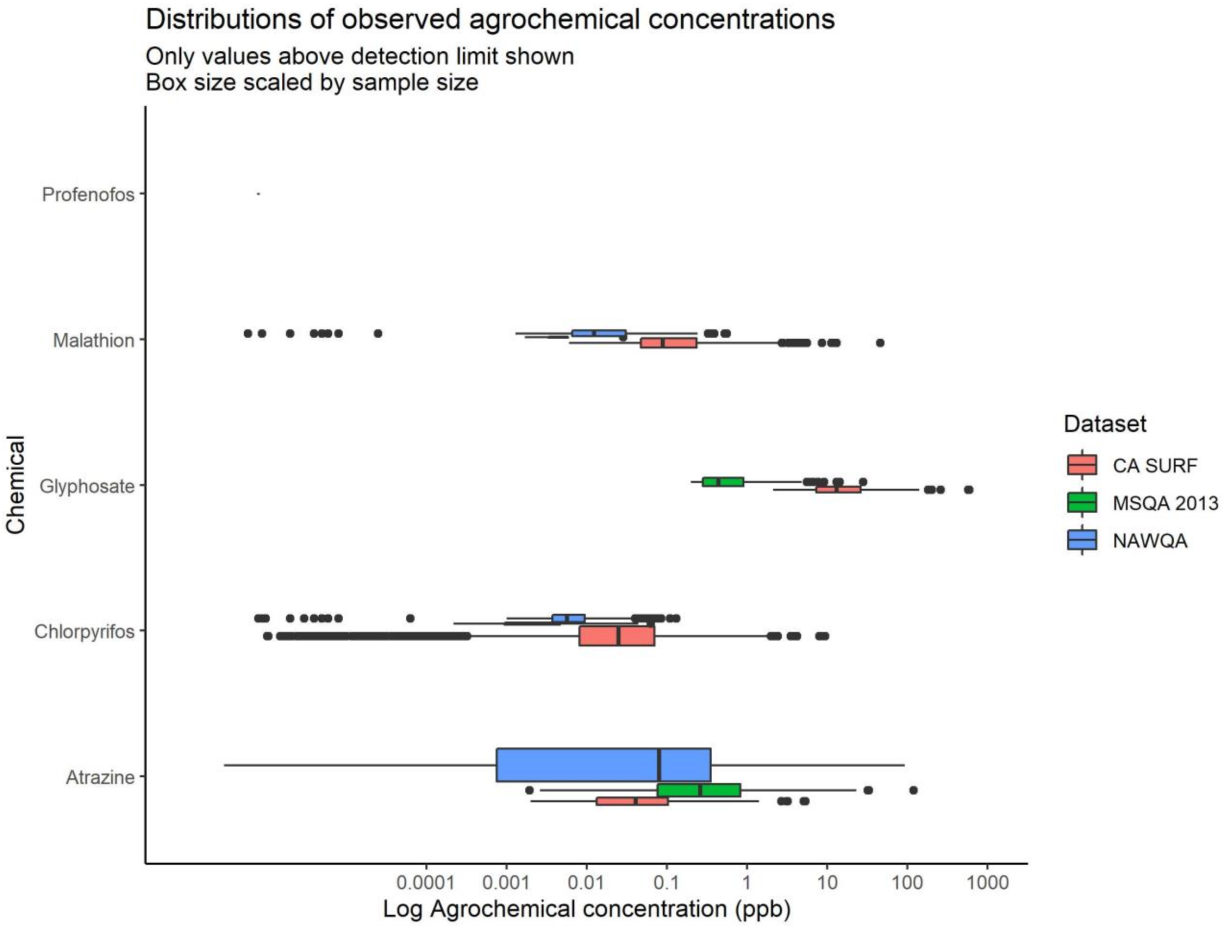

Figure S2: Distributions of agrochemical observations from pesticide monitoring databases used to derive POC_c

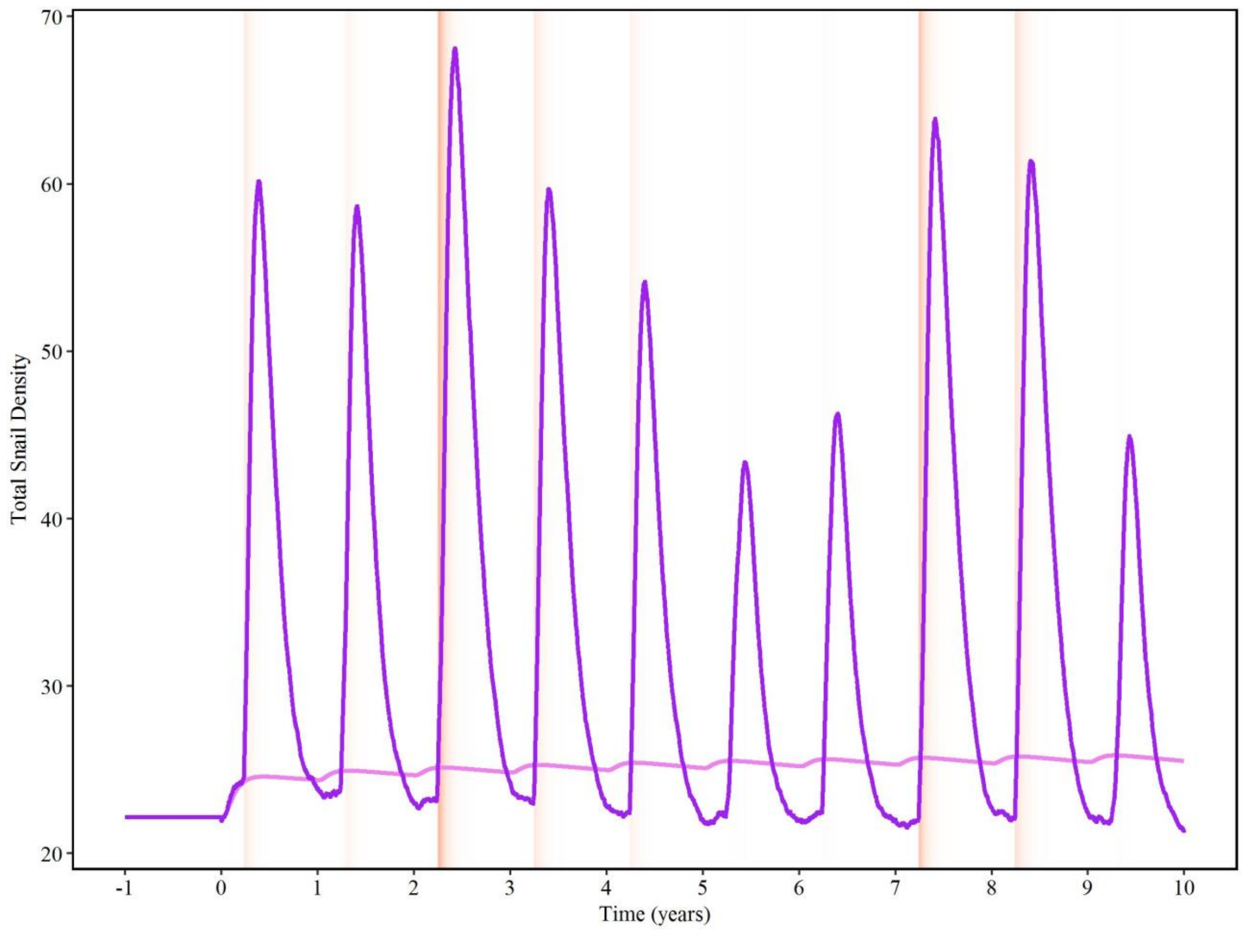

Figure S3: Snail population dynamics in MDA simulation with atrazine exposure. Only simulations including predators and annual MDA are shown for figure clarity, with dark purple line indicating snail population dynamics in the presence of atrazine pollution, and light purple lines indicating dynamics with no atrazine pollution. Background shading represents atrazine concentrations with darker shading indicating concentrations closer to $EEC_{atrazine}$

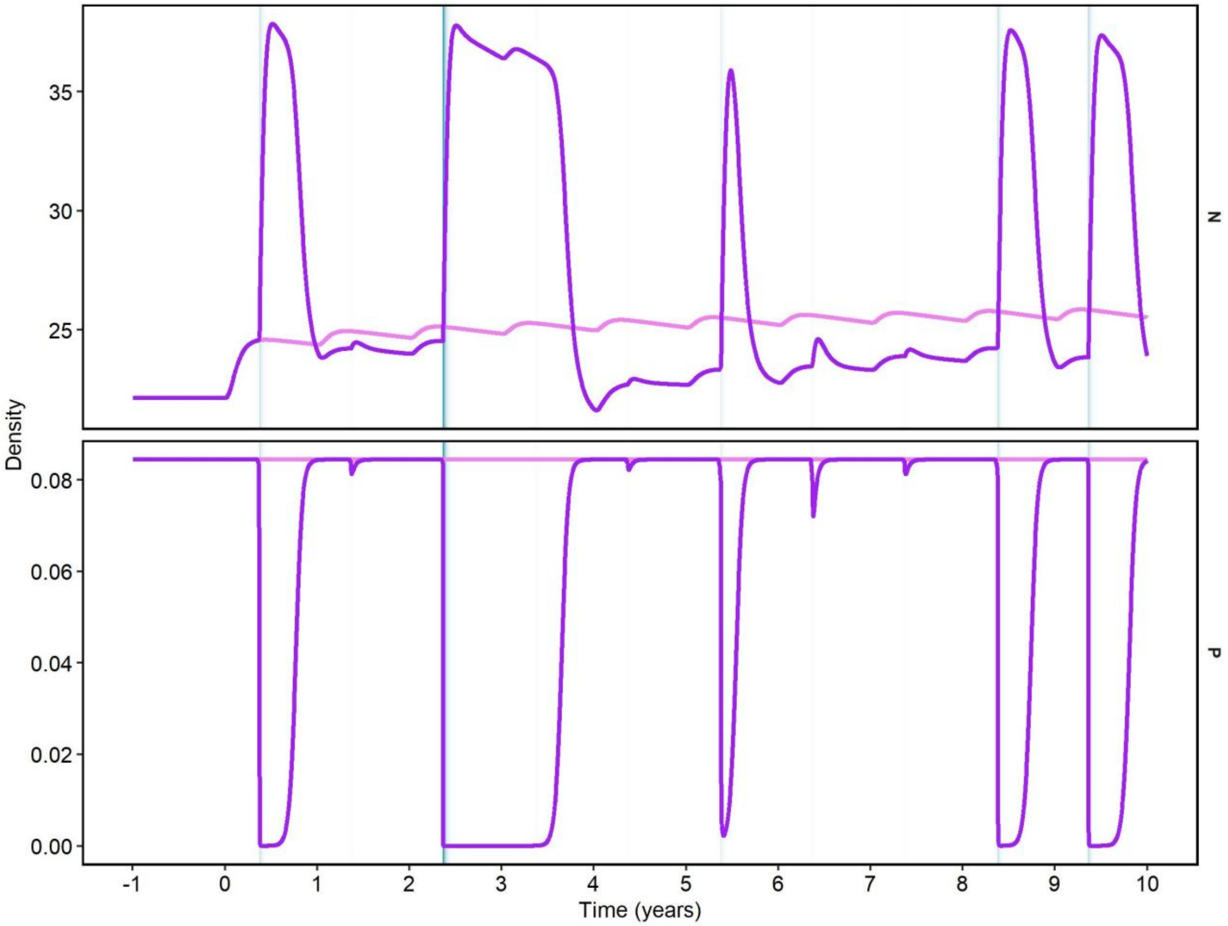

Figure S4: Snail (N) and predator (P) population dynamics in MDA simulation with profenofos exposure. Only simulations including predators and annual MDA are shown for figure clarity, with dark purple line indicating snail (top) and predator (bottom) population dynamics in the presence of profenofos pollution, and light purple lines indicating dynamics with no profenofos pollution. Background blue shading represents profenofos concentrations with darker shading indicating concentrations closer to $EEC_{profenofos}$. Predator populations are frequently decimated due to profenofos toxicity, leading to large increases in the snail population while the predator population rebounds.

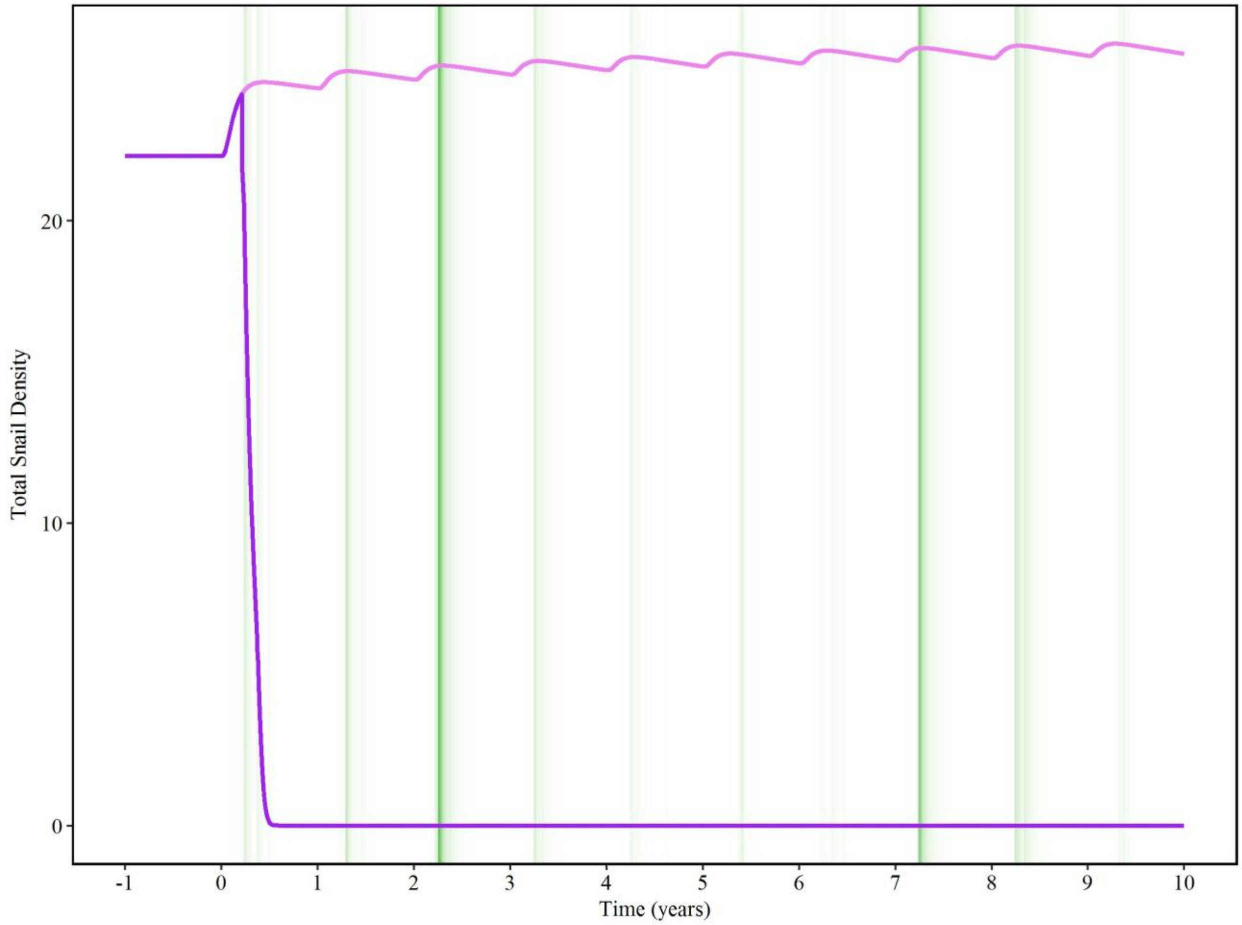

Figure S5: Snail population dynamics in MDA simulation with glyphosate exposure. Only simulations including predators and annual MDA are shown for figure clarity, with dark purple line indicating snail population dynamics in the presence of glyphosate pollution, and light purple lines indicating dynamics with no glyphosate pollution. Background shading represents glyphosate concentrations with darker shading indicating concentrations closer to $EEC_{glyphosate}$

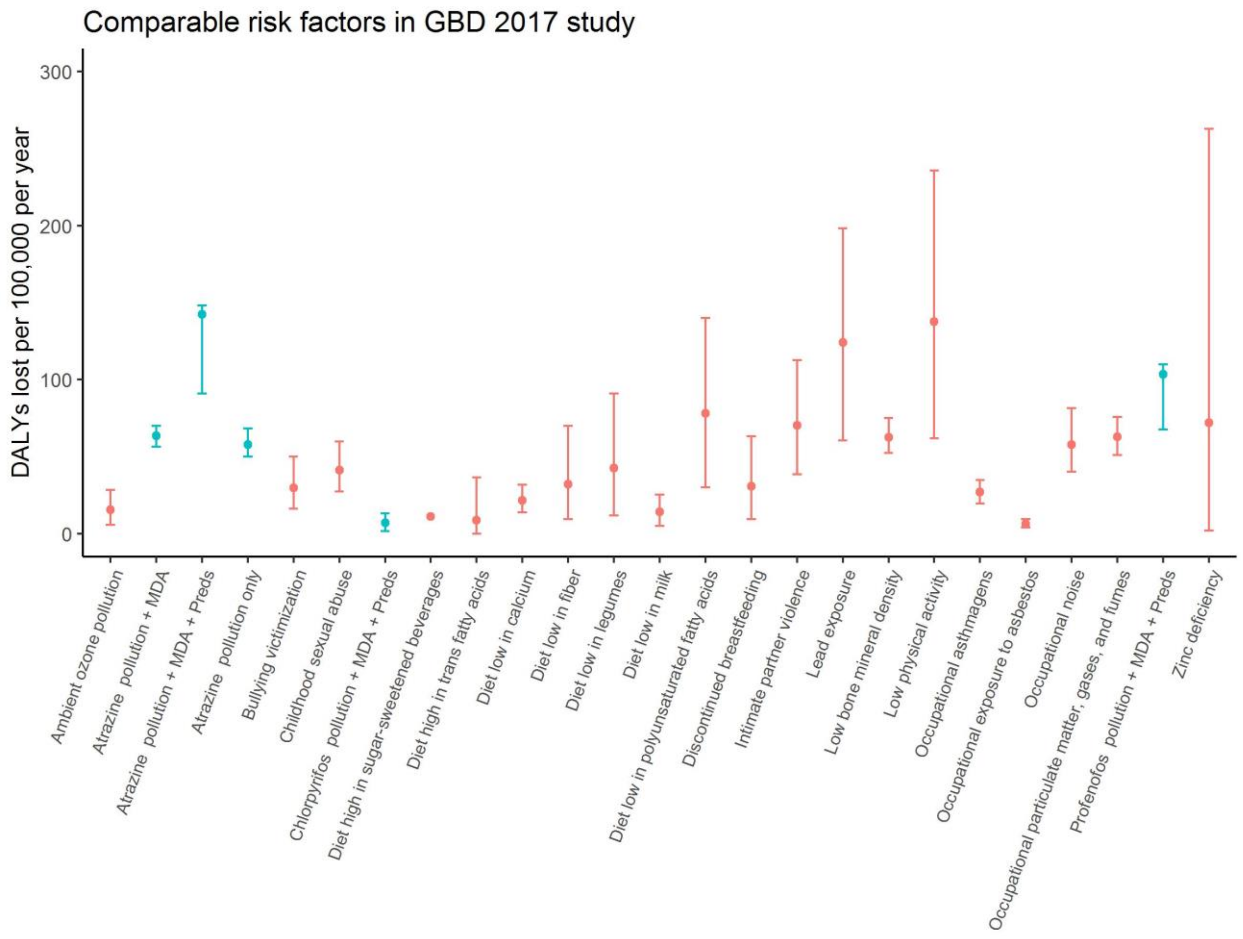

Figure S6: Comparison of DALYs attributable to agrochemical amplification of schistosomiasis transmission in different transmission scenarios (blue) with other exposures in the 2017 Global Burden of Disease study in Senegal (red). Error bars represent 95% confidence intervals for GBD exposures and the 95% sampling intervals from 1,000 MCMC simulations for agrochemical exposures, as described in the main text.

References

1 Abdel-Ghaffar F, Ahmed AK, Bakry F, Rabei I, Ibrahim A. The Impact of Three Herbicides on Biological and Histological Aspects of *Biomphalaria alexandrina* , Intermediate Host of *Schistosoma mansoni*. *Malacologia* 2016; **59**: 197–210.

2 Bajet CM, Kumar A, Calingacion MN, Narvacan TC. Toxicological assessment of pesticides used in the Pagsanjan-Lumban catchment to selected non-target aquatic organisms in Laguna Lake, Philippines. *Agric Water Manag* 2012; **106**: 42–9.

3 Bakry FA, Hasheesh WS, Hamdi SAH. Biological, biochemical, and molecular parameters of Helisoma duryi snails exposed to the pesticides Malathion and Deltamethrin. *Pestic Biochem Physiol* 2011; **101**: 86–92.

4 Bakry FA, Abdelsalam HA, Mahmoud MB, Hamdi SAH. Influence of Atrazine and Roundup pesticides on biochemical and molecular aspects of Biomphalaria alexandrina snails. *Pestic Biochem Physiol* 2012; **104**: 9–18.

5 Bakry FA, Eleiwa ME, Taha SA, Ismil SM. Comparative toxicity of Paraquat herbicide and some plant extracts in Lymnaea natalensis snails. *Toxicol Ind Health* 2016; **32**: 143–53.

6 Barbieri E, Moreira P, Luchini LA, Ruiz Hidalgo K, Muñoz A. Assessment of acute toxicity of carbofuran in Macrobrachium olfersii (Wiegmann, 1836) at different temperature levels. *Toxicol Ind Health* 2016; **32**: 7–14.

7 Baxter LR, Moore DL, Sibley PK, Solomon KR, Hanson ML. Atrazine does not affect algal biomass or snail populations in microcosm communities at environmentally relevant concentrations. *Environ Toxicol Chem* 2011; **30**: 1689–96.

8 Rohr JR, Halstead NT, Raffel TR. The herbicide atrazine, algae, and snail populations. *Environ Toxicol Chem* 2012; **31**: 973–4.

9 Benli AÇK, Sarıkaya R, Sepici-Dincel A, Selvi M, Şahin D, Erkoç F. Investigation of acute toxicity of (2,4-dichlorophenoxy)acetic acid (2,4-D) herbicide on crayfish (Astacus leptodactylus Esch. 1823). *Pestic Biochem Physiol* 2007; **88**: 296–9.

10 Bhavan PS, Geraldine P. Histopathology of the hepatopancreas and gills of the prawn Macrobrachium malcolmosonii exposed to endosulfan. *Aquat Toxicol* 2000; **50**: 331–9.

11 Browne AM, Moore PA. The Effects of Sublethal Levels of 2,4-Dichlorophenoxyacetic Acid Herbicide (2,4-D) on Feeding Behaviors of the Crayfish O. rusticus. *Arch Environ Contam Toxicol* 2014; **67**: 234–44.

12 Fornstrom CB, Landrum PF, Weisskopf CP, La Point TW. Effects of terbufos on juvenile red swamp crayfish (*Procambarus clarkii*): Differential routes of exposure. *Environ Toxicol Chem* 1997; **16**: 2514–20.

13 Griggs JL, Belden LK. Effects of Atrazine and Metolachlor on the Survivorship and Infectivity of Echinostoma trivolvis Trematode Cercariae. *Arch Environ Contam Toxicol* 2008; **54**: 195–202.

14 Gustafson KD, Belden JB, Bolek MG. Atrazine reduces the transmission of an amphibian trematode by altering snail and ostracod host-parasite interactions. *Parasitol Res* 2016; **115**: 1583–94.

15 Halstead NT, Civitello DJ, Rohr JR. Comparative toxicities of organophosphate and pyrethroid insecticides to aquatic macroarthropods. *Chemosphere* 2015; **135**: 265–71.

16 Halstead NT, Hoover CM, Arakala A, *et al.* Agrochemicals increase risk of human schistosomiasis by supporting higher densities of intermediate hosts. *Nat Commun* 2018; **9**: 837.

17 Hasheesh WS, Mohamed RT. Bioassay of two pesticides on Bulinus truncatus snails with emphasis on some biological and histological parameters. *Pestic Biochem Physiol* 2011; **100**: 1–6.

18 Hussein RM, Marie M-AS, Afifi F, El-Deeb A, Sayed S, Sayed M. Effects of Three Inorganic Fertilizers on

the Biology and Histopathology of infected Biomphalaria alexandrina snails. http://www.rjpbcs.com/pdf/2016_7(4)/[320].pdf (accessed April 21, 2017).

19 Ibrahim WLF, Furu P, Ibrahim AM, Christensen NO. Effect of the organophosphorous insecticide, chlorpyrifos (Dursban), on growth, fecundity, and mortality of biomphalaria alexandrina and on the production of schistosoma mansoni cercariae in the snail. *J Helminthol* 1992; **66**: 79–88.

20 Johnson PTJ, Chase JM, Dosch KL, *et al.* Aquatic eutrophication promotes pathogenic infection in amphibians. *Proc Natl Acad Sci* 2007; **104**: 15781–6.

21 Koprivnikar J, Forbes MR, Baker RL. EFFECTS OF ATRAZINE ON CERCARIAL LONGEVITY, ACTIVITY, AND INFECTIVITY. *J Parasitol* 2006; **92**: 306–11.

22 Kristoff G, Cacciatore LC, Guerrero NR V, Cochon AC. Effects of the organophosphate insecticide azinphos-methyl on the reproduction and cholinesterase activity of Biomphalaria glabrata. *Chemosphere* 2011; **84**: 585–91.

23 Lavarías S, García CF. Acute toxicity of organophosphate fenitrothion on biomarkers in prawn Palaemonetes argentinus (Crustacea: Palaemonidae). *Environ Monit Assess* 2015; **187**: 65.

24 Leung TS, Naqvi SM, Naqvi NZ. Paraquat toxicity to Louisiana crayfish (Procambarus clarkii). *Bull Environ Contam Toxicol* 1980; **25**: 465–9.

25 Mohamed AM, El-Emam MA, Osman GY, Abdel-Hamid H, Ali REM. Effect of Basudin, Selecron and the phytoalkaloid Colchicine (pesticides) on biological and molecular parameters of Biomphalaria alexandrina snails. *Pestic Biochem Physiol* 2012; **102**: 68–78.

26 Monde C, Syampungani S, Van den Brink PJ. Effects of Endosulfan on Predator–Prey Interactions Between Catfish and Schistosoma Host Snails. *Arch Environ Contam Toxicol* 2016; **71**: 257–66.

27 Monte TC de C, Garcia J, Gentile R, *et al.* In vivo and in vitro effects of the herbicide Roundup® on developmental stages of the trematode Echinostoma paraensei. *Exp Parasitol* 2016; **169**: 43–50.

28 Naqvi SM, Leung TS. Trifluralin and oryzalin herbicides toxicities to juvenile crawfish (Procambarus clarkii) and mosquitofish (Gambusia affinis). *Bull Environ Contam Toxicol* 1983; **31**: 304–8.

29 Naqvi SM, Hawkins R, Naqvi NH. Mortality response and LC50 values for juvenile and adult crayfish, Procambarus clarkii exposed to Thiodan (insecticide), Treflan, MSMA, Oust (herbicides) and Cutrine-Plus (algicide). *Environ Pollut* 1987; **48**: 275–83.

30 Oliveira-Filho EC, Grisolia CK, Paumgartten FJR. Effects of endosulfan and ethanol on the reproduction of the snail Biomphalaria tenagophila: A multigeneration study. *Chemosphere* 2009; **75**: 398–404.

31 Omkar, Murti R. Toxicity of Some Pesticides to the Freshwater Prawn, Macrobrachium dayanum (Henderson) (Decapoda, Caridea). Crustaceana. ; **49**: 1–6.

32 Omkar, Shukla GS. Toxicity of Insecticides to Macrobrachium lamarrei (H. Milne Edwards) (Decapoda, Palaemonidae). Crustaceana. ; **48**: 1–5.

33 Omran NE, Salama WM. The endocrine disruptor effect of the herbicides atrazine and glyphosate on Biomphalaria alexandrina snails. *Toxicol Ind Health* 2016; **32**: 656–65.

34 Ragab FMA, Shoukry NM. Influence of certain fertilizers on the activity of some molluscicides against Biomphalaria alexandrina and Lymnaea natalensis snails. *J Egypt Soc Parasitol* 2006; **36**: 959–77.

35 Revathi P, Munuswamy N. Effect of tributyltin on the early embryonic development in the freshwater prawn Macrobrachium rosenbergii (De Man). *Chemosphere* 2010; **79**: 922–7.

36 Rohr JR, Schotthoefer AM, Raffel TR, *et al.* Agrochemicals increase trematode infections in a declining amphibian species. *Nature* 2008; **455**: 1235–9.

37 Rohr JR, Raffel TR, Sessions SK, Hudson PJ. Understanding the net effects of pesticides on amphibian

trematode infections. *Ecol Appl* 2008; **18**: 1743–53.

38 Sarojini R, Nagabhushanam R, Avelin Mary S. Effect of fenitrothion on reproduction of the freshwater prawn Macrobrachium lamerrii. *Ecotoxicol Environ Saf* 1986; **11**: 243–50.

39 Satapornvanit K, Baird DJ, Little DC. Laboratory toxicity test and post-exposure feeding inhibition using the giant freshwater prawn Macrobrachium rosenbergii. *Chemosphere* 2009; **74**: 1209–15.

40 Tantawy AA. Effect of two herbicides on some biological and biochemical parameters of Biomphalaria alexandrina. *J Egypt Soc Parasitol* 2002; **32**: 837–47.

41 Tchounwou PB, Englande AJ, Malek EA, Anderson AC, Abdelghani AA. The effects of bayluscide and malathion on the survival of Schistosoma mansoni miracidia. *J Environ Sci Heal Part BOnline) J Abdelghani J Environ Sci Heal Part B J ENVIRON SCI Heal* 1991; **26**: 360–1234.

42 Tchounwou PB, Englande AJ, Malek EA, Anderson AC, Abdelghani AA. The effects of ammonium sulphate and urea upon egg hatching and miracidial survival of Schistosoma mansoni. *J Environ Sci Heal Part B* 1991; **26**: 241–57.

43 Tchounwou PB, Englande AJ, Malek EA, Greer GJ, Anderson AC. The effects of bayluscide and malathion on the mortality and infectivity ofSchistosoma mansoni cercariae. *Environ Toxicol Water Qual* 1992; **7**: 107–17.

44 Young DF. The Variable Volume Water Model Revision A. Washington, DC, 2016.

45 Burns LA. Exposure Analysis Modeling Systems (EXAMS) User Manual and System Documentation. Research Triangle Park, NC, 2000 https://cfpub.epa.gov/si/si_public_record_report.cfm?dirEntryId=64143&Lab=NERL.

46 Young DF. Pesticide in Water Calculator User Manual. Washington, DC, 2015 https://www.epa.gov/sites/production/files/2015-12/documents/pwc_user_manual_12-8-15.pdf.

47 Ritz C, Baty F, Streibig JC, Gerhard D, Baun A, Nyholm N. Dose-Response Analysis Using R. *PLoS One* 2015; **10**: e0146021.

48 Sokolow SH, Huttinger E, Jouanard N, *et al.* Reduced transmission of human schistosomiasis after restoration of a native river prawn that preys on the snail intermediate host. *Proc Natl Acad Sci U S A* 2015; **112**: 9650–5.

49 Anderson RMRM, May RMRM. Infectious Diseases of Humans. New York, NY, NY: Oxford University Press, 1991.

50 Macdonald G. The dynamics of helminth infections, with special reference to schistosomes. *Trans R Soc Trop Med Hyg* 1965; **59**: 489–506.

51 Lafferty KD, Kuris AM. Parasitic castration: the evolution and ecology of body snatchers. *Trends Parasitol* 2009; **25**: 564–72.

52 Holling CS. The Components of Predation as Revealed by a Study of Small-Mammal Predation of the European Pine Sawfly. *Can Entomol* 1959; **91**: 293–320.

53 May RM. Togetherness among Schistosomes: its effects on the dynamics of the infection. *Math Biosci* 1977; **35**: 301–43.

54 Cervantes-Santiago E, Hernández-Vergara MP, Pérez-Rostro CI, Olvera-Novoa MA. Reproductive performance of the crayfish *Procambarus* (*Austrocambarus*) *acanthophorus* Villalobos 1948 under controlled conditions. *Aquaculture* 2010; **308**: 66–70.

55 Real LA. The Kinetics of Functional Response. *Am Nat* 1977; **111**: 289–300.

56 Hofkin B V, Mkoji GM, Koech DK, Loker ES. Control of schistosome-transmitting snails in Kenya by the North American crayfish Procambarus clarkii. *Am J Trop Med Hyg* 1991; **45**: 339–44.

57 Goddard MJ, Jordan P. On the longevity of *Schistosoma mansoni* in man on St. Lucia, West Indies. *Trans R Soc Trop Med Hyg* 1980; **74**: 185–91.

58 Woolhouse MEJ, Chandiwana SK. Population biology of the freshwater snail *Bulinus globosus* in the Zimbabwe highveld. *J Appl Ecol* 1990; **27**: 41–59.

59 Pflüger W, Roushdy MZ, El Emam M. The prepatent period and cercarial production of Schistosoma haematobium in Bulinus truncatus (Egyptian field strains) at different constant temperatures. *Zeitschrift für Parasitenkd (Berlin, Ger* 1984; **70**: 95–103.

60 Sokolow SH, Lafferty KD, Kuris AM. Regulation of laboratory populations of snails (*Biomphalaria* and *Bulinus* spp.) by river prawns, *Macrobrachium* spp. (Decapoda, Palaemonidae): implications for control of schistosomiasis. *Acta Trop* 2014; **132**: 64–74.

61 Arakala A, Hoover CM, Marshall JM, *et al.* Estimating the elimination feasibility in the ‘end game’ of control efforts for parasites subjected to regular mass drug administration: Methods and their application to schistosomiasis. *PLoS Negl Trop Dis* 2018; **12**: e0006794.

62 Truscott JE, Gurarie D, Alsallaq R, *et al.* A comparison of two mathematical models of the impact of mass drug administration on the transmission and control of schistosomiasis. *Epidemics* 2017; **18**: 29–37.

63 Lo NC, Gurarie D, Yoon N, *et al.* Impact and cost-effectiveness of snail control to achieve disease control targets for schistosomiasis. *Proc Natl Acad Sci U S A* 2018; **115**: E584–91.

64 King CH, Dickman K, Tisch DJ. Reassessment of the cost of chronic helmintic infection: a meta-analysis of disability-related outcomes in endemic schistosomiasis. *Lancet* 2005; **365**: 1561–9.

65 Hoover CM, Sokolow SH, Kemp J, *et al.* Modelled effects of prawn aquaculture on poverty alleviation and schistosomiasis control. *Nat Sustain* 2019; **2**: 611–20.
